# Lsd1 regulates skeletal muscle regeneration and directs the fate of satellite cells

**DOI:** 10.1038/s41467-017-02740-5

**Published:** 2018-01-25

**Authors:** Milica Tosic, Anita Allen, Dominica Willmann, Christoph Lepper, Johnny Kim, Delphine Duteil, Roland Schüle

**Affiliations:** 1 Urologische Klinik und Zentrale Klinische Forschung, Universitätsklinikum Freiburg, Medizinische Fakultät, Albert-Ludwigs-University Freiburg, Breisacherstrasse 66, 79106 Freiburg, Germany; 2 Department of Embryology, Carnegie Institution, 3520 San Martin Drive, Baltimore, MD 21218 USA; 3 Department of Cardiac Development and Remodeling, Max Planck Institute for Heart and Lung Research, Ludwigstraße 43, 61231 Bad Nauheim, Germany; 4 BIOSS Centre for Biological Signalling Studies, Albert-Ludwigs-University, 79104 Freiburg, Germany; 5 Deutsches Konsortium für Translationale Krebsforschung (DKTK), Freiburg, 79106 Germany; 6 K-metics GmbH, Breisacherstrasse 66, 79106 Freiburg, Germany

## Abstract

Satellite cells are muscle stem cells required for muscle regeneration upon damage. Of note, satellite cells are bipotent and have the capacity to differentiate not only into skeletal myocytes, but also into brown adipocytes. Epigenetic mechanisms regulating fate decision and differentiation of satellite cells during muscle regeneration are not yet fully understood. Here, we show that elevated levels of lysine-specific demethylase 1 (Kdm1a, also known as Lsd1) have a beneficial effect on muscle regeneration and recovery after injury, since Lsd1 directly regulates key myogenic transcription factor genes. Importantly, selective Lsd1 ablation or inhibition in Pax7-positive satellite cells, not only delays muscle regeneration, but changes cell fate towards brown adipocytes. Lsd1 prevents brown adipocyte differentiation of satellite cells by repressing expression of the novel pro-adipogenic transcription factor Glis1. Together, downregulation of Glis1 and upregulation of the muscle-specific transcription program ensure physiological muscle regeneration.

## Abstract (web summary)

Satellite cells can differentiate both into myocytes and brown adipocytes. Here, the authors show that the histone demethylase Lsd1 prevents adipogenic differentiation of satellite cells by repressing expression of Glis1, and that its ablation changes satellite cell fate towards brown adipocytes and delays muscle regeneration in mice.

## Introduction

Muscle damage occurs as a consequence of disease, ischemia, and injury induced by trauma or excessive exercise^1^. In adult skeletal muscle, stem cells required for muscle regeneration reside underneath the basal lamina of individual muscle fibers and are termed satellite cells^2^. Under physiological conditions, satellite cells are in a quiescent state and express the transcription factor paired box 7 (Pax7)^3^. Upon injury, myofibers undergo degeneration accompanied with inflammatory cell infiltration, followed by massive and rapid activation, proliferation, and myogenic differentiation of satellite cells^4^.

Adult muscle regeneration resembles embryonic muscle development, since it requires activation of the muscle regulatory gene network^5^. The transcription factors Pax7 and its paralog Pax3 activate the expression of myogenic factor 5 (*Myf5)* and myogenic differentiation 1 (*Myod1)*. Myf5 and Myod1 are required for myoblast specification^6,7^, while downstream transcription factor myogenin (Myog) governs differentiation into myotubes^8^. The family of myocyte enhancer factor 2 (Mef2) proteins acts in cooperation with Myog and Myod1 to drive terminal muscle differentiation^9,10^. During this process, mononuclear myocytes fuse to form mature multinucleated muscle fibers expressing myosin heavy chain (*Myh*), myosin light chain (*Myl*) isoforms, and muscle creatine kinase (*Ckm*)^11^. Recently, important roles of epigenetic chromatin remodelers and microRNA in muscle regeneration have been discovered^12,13^. Nevertheless, the molecular biology of these regulatory processes is not yet fully understood.

Former studies have shown that satellite cells are bipotent and can yield not only skeletal myocytes, but also brown adipocytes, when differentiated under pro-adipogenic conditions^14,15^. Moreover, appearance of brown adipocytes in murine skeletal muscle can be induced by cold exposure^14^. Fate determination of satellite cells is, at least in part, controlled by a myogenic microRNA, miR-133, that represses brown fat determining zinc-finger protein Prdm16^15^ and consequently inhibits differentiation into brown adipocytes^14,16^.

Lysine-specific demethylase 1 (Lsd1) is an epigenetic enzyme involved in transcriptional repression or activation of genes through demethylation of mono- and dimethylated histone H3 at lysine 4 (H3K4me1 and H3K4me2) and lysine 9 (H3K9me2 and H3K9me1), respectively^17–19^. Previous studies indicated that Lsd1 plays a role in muscle differentiation^20–22^, since Lsd1 knockdown in C2C12 skeletal myoblasts impairs myogenesis^22^. In C2C12 cells, Lsd1 interacts with Myod1 and Mef2d to remove the repressive histone mark H3K9me2 from *Myog* and *Ckm* promoters^22^. Lsd1 is also required for the timely expression of Myod1 in limb buds of E11.5 mouse embryos, through the regulation of Myod1 core enhancer element^21^. Despite the described function of Lsd1 in skeletal muscle differentiation, its role in muscle regeneration has been poorly characterized.

In addition to its role in skeletal muscle, several studies implicated Lsd1 in the differentiation of white and beige adipocytes in vitro^23^ and in vivo^24^. Consistently, in mouse embryos it was demonstrated that Lsd1 promotes development of the brown adipose tissue (BAT)^25^. Since Lsd1 is involved in both myogenesis and adipogenesis, we questioned whether it would also play a role in fate decision of bipotent satellite cells.

In this study, we show that Lsd1 promotes muscle regeneration by increasing the differentiation capacity of satellite cells through direct regulation of muscle-specific genes. Vice versa, Lsd1 ablation or inhibition delays muscle regeneration and results in infiltration of satellite cell-derived brown adipocytes into muscle fibers. Our work implicates that Lsd1 is indispensable for fate decision of satellite cells and acts to repress their adipogenic potential by downregulating the newly identified pro-adipogenic transcription factor Glis1.

## Results

### Lsd1 regulates skeletal muscle regeneration

**Fig. 1:**
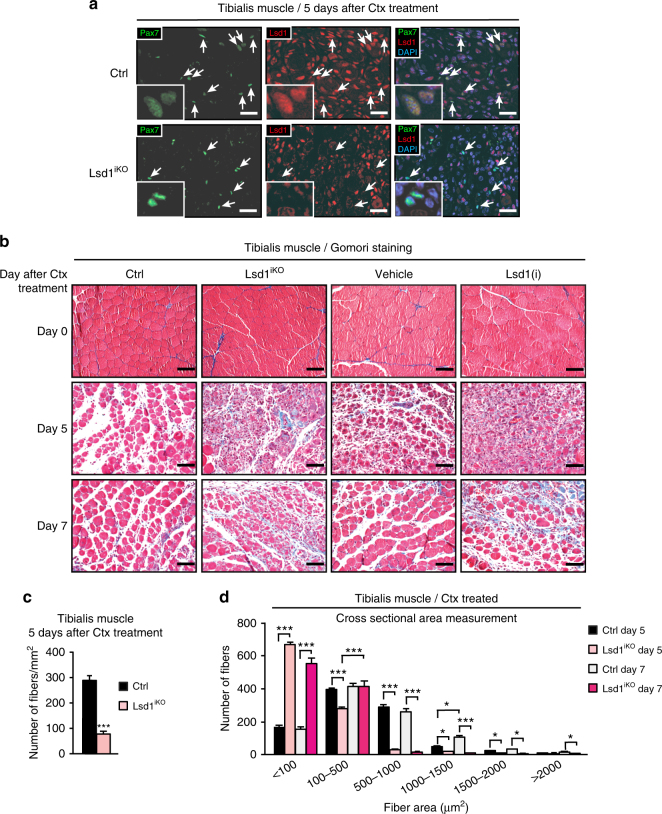
Lsd1 ablation or inhibition delays skeletal muscle regeneration. **a** Immunofluorescence assay using antibodies directed against paired box 7 (Pax7, green) and lysine-specific demethylase 1 (Lsd1, red) on tibialis muscle sections of control mice (Ctrl) or mice with selective Lsd1 ablation in Pax7-positive satellite cells (Lsd1^iKO^) 5 days after cardiotoxin (Ctx) treatment. Nuclei were stained with DAPI (blue). Arrows indicate that Lsd1 is expressed in Pax7-positive satellite cells of control mice, whereas it is ablated from Lsd1^iKO^ Pax7-positive satellite cells. **b** Gomori staining of representative tibialis muscle sections from Ctrl, Lsd1^iKO^ mice, and wild-type mice treated with vehicle or Lsd1 inhibitor [Lsd1(i)], 0, 5, and 7 days after cardiotoxin (Ctx) injection. **c**, **d** Analyses of regenerating centronuclear fibers in Ctrl and Lsd1^iKO^ mice 5 or 7 days after Ctx treatment. **c** Number of fibers per area (mm^2^). Significance was calculated by two-tailed Student’s *t*-test. **d** Cross-sectional area (CSA) measurement of fibers. Significance was calculated by two-way analysis of variance (ANOVA) test. (**c**,** d**: *n* = 5; mean + SEM, *p < 0.05, **p < 0.01, ***p < 0.001; scale bars: **a** 50 µm, **b** 100 µm)

Since loss of Lsd1 in C2C12 myoblasts impairs myogenesis^22^, we hypothesized that Lsd1 might play a role in skeletal muscle regeneration. To determine whether Lsd1 protein is expressed during muscle regeneration, we induced muscle damage by injecting cardiotoxin (Ctx) into the murine tibialis anterior muscle and performed immunofluorescence analyses. We found that Lsd1 is expressed in the nuclei of Pax7-positive satellite cells (Fig. 1a) as well as in the centronuclei of regenerating muscle fibers (Supplementary Fig. 1a).

To assess potential roles of Lsd1 during skeletal muscle regeneration, we generated mice with Lsd1 ablation (hereafter named Lsd1^iKO^ mice, Fig. 1a) or mice expressing an enzymatically inactive Lsd1 mutant from the endogenous *Lsd1* promoter (hereafter named Lsd1^iKI^ mice, Supplementary Figs. 1b and 9a) selectively in satellite cells. This was accomplished by crossing mice expressing tamoxifen (Tam) inducible *Cre recombinase* under the control of the *Pax7* promoter (Pax7^Cre/ERT2^)^26^ with mice harboring conditional *Lsd1* alleles (Lsd1^fl/fl^)^27^ or conditional *Lsd1* mutant knock-in alleles (Lsd1^KI/KI^)^28^, respectively, and subsequently treating them with Tam. Lsd1^iKO^ and Lsd1^iKI^ mice were also crossed with mice harboring a Cre-dependent green fluorescent protein (GFP) reporter transgene^29^, which allowed us to trace the fate of satellite cells. Furthermore, we treated wild-type mice the highly specific, nanomolar affinity Lsd1 inhibitor ORY-1001^30^ [referred to as Lsd1(i) mice] to investigate the effect of chemical Lsd1 inactivation on muscle regeneration.

Regeneration efficiency was evaluated by observing fiber morphology and fibrosis on Gomori-stained sections 0, 5, and 7 days after Ctx treatment (Fig. 1b). Untreated tibialis muscle of Lsd1^iKO^ mice and control littermates displayed no difference in morphology and fiber size distribution (Supplementary Fig. 1c, d), as demonstrated by dystrophin (Dmd) staining and determination of fiber cross-sectional area (CSA). In contrast, muscle regeneration was strongly retarded in Lsd1^iKO^ compared to control mice, since at day 5 and 7 post-Ctx treatment Lsd1-ablated muscles displayed increased fibrosis and fewer regenerating fibers (Fig. 1b, c). CSA measurement showed that the most abundant fibers in Lsd1^iKO^ muscle had a surface area smaller than 100 μm^2^, whereas larger fibers were significantly less represented than in control (Ctrl) muscle (Fig. 1d). Decreased regenerative capacity was also observed in Lsd1^iKI^ and Lsd1(i) mice, demonstrating that Lsd1 demethylase activity is required for proper muscle regeneration (Fig. 1b and Supplementary Fig. 1c, e–h). Of note, injecting Lsd1 inhibitor or vehicle without Ctx caused negligible muscle damage (Supplementary Fig. 1i). Together, these data indicate that Lsd1 ablation or inhibition delays the process of skeletal muscle regeneration.

**Fig. 2:**
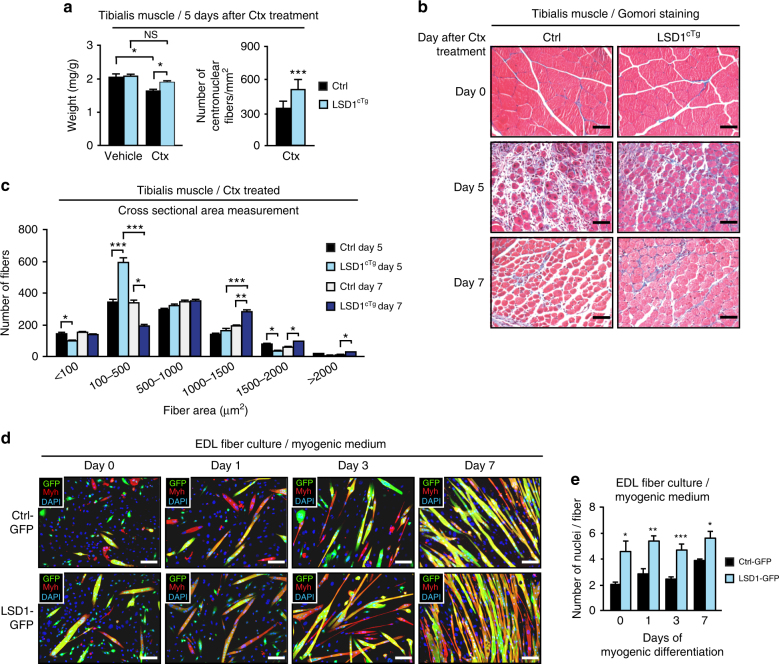
Lsd1 overexpression accelerates skeletal muscle regeneration. **a** Tibialis muscle mass normalized to body mass of control mice (Ctrl) or mice overexpressing LSD1 selectively in Myf5-positive muscle precursors (LSD1^cTg^) 5 days after Ctx or vehicle injection (left panel). Number of regenerating centronuclear fibers per area (mm^2^) in Ctrl and LSD1^cTg^ mice 5 days after Ctx treatment (right panel). Significance was calculated by two-way ANOVA test. **b** Gomori staining of representative tibialis muscle sections from Ctrl and LSD1^cTg^ mice 0, 5, and 7 days after Ctx treatment. **c** CSA measurement of regenerating centronuclear muscle fibers from Ctrl and LSD1^cTg^ mice 5 and 7 days after Ctx treatment. Significance was calculated by two-way ANOVA test. **d** Immunofluorescence assay using antibodies directed against pan-myosin heavy chain (Myh, red) and green fluorescent protein (GFP, green) on control (Ctrl-GFP) and LSD1 overexpressing (LSD1-GFP) satellite cells isolated from extensor digitorum longus (EDL) fiber culture differentiated in myogenic medium for 0, 1, 3, or 7 days. Nuclei were stained with DAPI (blue). **e** Quantification of the nuclei per fiber shown in immunofluorescence assay in **d**. Significance was calculated by two-tailed Student’s *t*-test. (**a**, **c**: *n* = 5, **e**: *n *= 3; mean + SEM, NS = non-significant, *p < 0.05, **p < 0.01, ***p < 0.001; scale bars: **b**, **d** 100 µm)

To investigate whether elevated Lsd1 levels would ameliorate skeletal muscle regeneration, we engineered transgenic mice in which expression of flag-tagged human *LSD1* is under the control of chicken *ß-actin* promoter^28^. Expression of the transgene requires Cre-mediated excision of a translational stop cassette. Crossing these transgenic mice with the *Myf5-Cre* deleter strain^31^, we obtained mice, in which LSD1 overexpression is induced in myoblast precursors (hereafter named Lsd1^cTg^ mice). Western blot analyses showed ~2–3-fold overexpression of LSD1 in the muscle of LSD1^c^^Tg^ mice compared to Ctrl (Supplementary Figs. 2a and 9b). Ctx-induced muscle injury was accompanied with a decrease in muscle weight, which was significantly abrogated in LSD1^cTg^ mice (Fig. 2a). At day 5 post Ctx treatment, we observed a higher number of regenerating centronuclear fibers in LSD1^cTg^ mice compared to Ctrl mice (Fig. 2a, b). Opposite to our observations in Lsd1^iKO^ mice, Lsd1^cTg^ mice had decreased numbers of fibers with small surface area (<100 μm^2^), but increased numbers of fibers with a surface area 100–500 of μm^2^ (Fig. 2c). At day 7 post treatment, fibers with larger surface area (>1000 μm^2^) prevailed in LSD1^cTg^ mice, indicating faster regeneration (Fig. 2c). Of note, morphology and fiber size distribution of untreated tibialis muscle from LSD1^cTg^ mice was not different from that of their control littermates (Ctrl) (Supplementary Fig. 2b, c). Together, our data indicate that higher Lsd1 levels in myoblast precursors accelerate muscle regeneration upon injury.

To confirm that an increased differentiation capacity of satellite cells is responsible for faster muscle regeneration in vivo, we isolated satellite cells from myofibers of wild-type extensor digitorum longus (EDL) muscle^32^, overexpressed LSD1 by adenoviral infection, and differentiated them in myogenic medium for 0, 1, 3, or 7 days. Constructs used for adenoviral infection additionally harbored a GFP cassette, allowing us to trace transduced cells. LSD1 overexpressing satellite cells (LSD1-GFP) had higher differentiation capacity than control cells (Ctrl-GFP), since they yielded myofibers with more centronuclei per fiber (Fig. 2d, e and Supplementary Fig. 2d), more polynucleated fibers, and less mononucleated myocytes (Supplementary Fig. 2e). In addition, qRT-PCR analyses showed elevated mRNA levels of the myogenic markers *Myod1, Myog, Mef2a, Mef2c, Mef2d, Ckm, Myh3, Myh8*, and *Myl1* compared to Ctrl-GFP myofibers (Supplementary Fig. 2f). Together, our data show that elevated LSD1 levels in muscle precursors lead to faster muscle recovery, while Lsd1 ablation or inhibition delays skeletal muscle regeneration.

### Loss of Lsd1 favors brown adipocyte differentiation

**Fig. 3:**
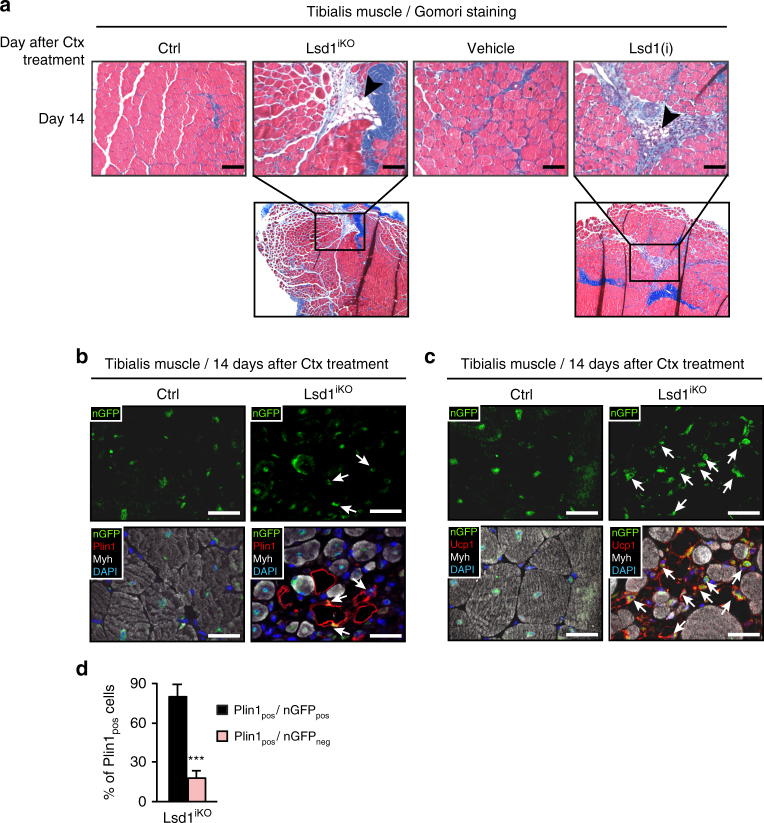
Loss of Lsd1 favors differentiation of satellite cells into brown adipocytes. **a**–**d** Representative tibialis muscle sections from Ctrl, Lsd1^iKO^, and Lsd1(i) mice 14 days after Ctx injection. **a** Gomori staining. Arrowheads indicate fat accumulation. **b**, **c** Immunofluorescence assays using antibodies directed against GFP (green), Myh (white), and **b** perilipin 1 (Plin1, red) or **c** uncoupling protein 1 (Ucp1, red) as indicated. Nuclei were stained with DAPI (blue). Arrows indicate adipocytes that express nuclear GFP (nGFP) and therefore originate from satellite cells. **d** Percentage of Plin1-positive cells that express or do not express nGFP. Significance was calculated by two-tailed Student’s *t*-test. (**d**: *n* = 6; mean + SEM, ***p < 0.001; scale bars: **a** 100 µm, **b**, **c** 50 µm)

Surprisingly, in Lsd1^iKO^ muscle 14 days after Ctx injury we noticed cells with an adipocyte-like morphology, intermingled with regenerating muscle fibers (Fig. 3a). These cells expressed perilipin 1 (Plin1) (Fig. 3b), coating lipid droplets, and uncoupling protein Ucp1 exclusively present in the inner mitochondrial membrane of functional brown adipocytes (Fig. 3c). Similar brown adipocyte accumulations were present in the tibialis muscle of Lsd1^iKI^ and Lsd1(i) mice, demonstrating that loss of Lsd1 demethylase activity is responsible for the observed phenotype (Fig. 3a–c and Supplementary Fig. 3a–c). Such a cell population was not observed in Ctx-treated Ctrl muscle (Fig. 3a–c and Supplementary Fig. 3a–c) or untreated muscle (Fig. 1b and Supplementary Fig. 1f). Since satellite cells are multipotent cells that can differentiate into myocytes and brown adipocytes^14^, we speculated that loss of Lsd1 in regenerating muscle might promote brown adipocyte differentiation. Fate tracing of Pax7-positive satellite cells in Lsd1^iKO^ and Lsd1^iKI^ muscle using the GFP reporter confirmed that Plin1-positive cells originated from satellite cells, since majority of these cells expressed nuclear GFP signal (nGFP) (Fig. 3b–d and Supplementary Fig. 3b–d). Of note, brown adipocytes were still observable in muscle one month after Ctx injection (Supplementary Fig. 3e). Together, our findings show that loss of Lsd1 or inhibition of its enzymatic activity in satellite cells favors a fate switch towards brown adipocytes.

**Fig. 4:**
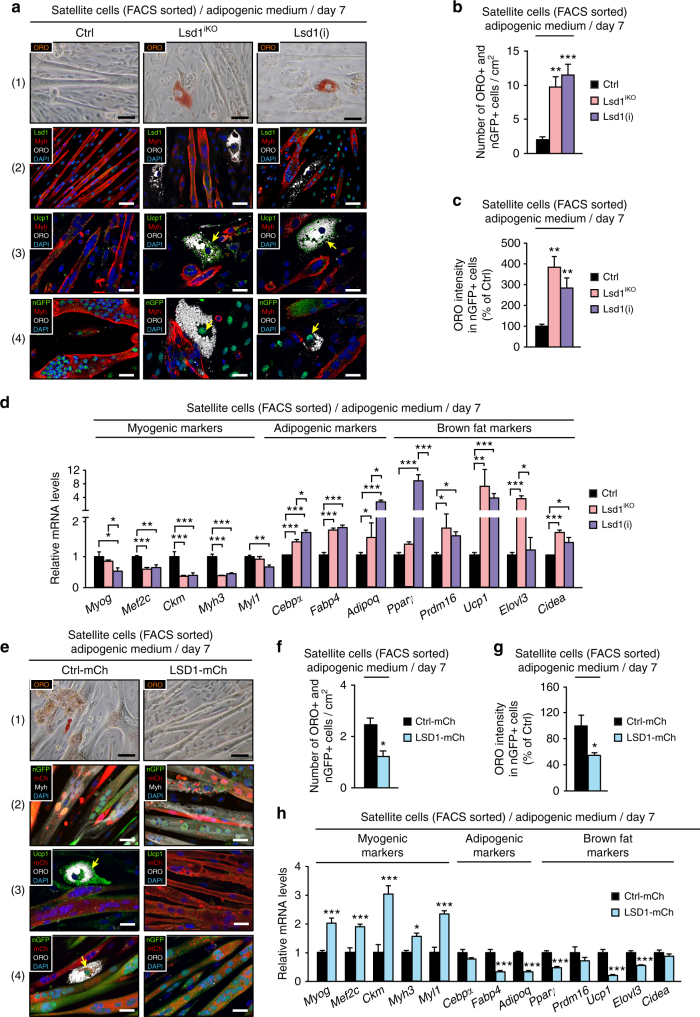
Lsd1 represses brown adipogenesis in satellite cells. **a**–**d** Analyses of FACS sorted Ctrl, Lsd1^iKO^, and Lsd1(i)-treated satellite cells differentiated for 7 days in adipogenic medium. **a** Oil red O (ORO) staining (1) and immunofluorescence assays using antibodies directed against: Lsd1 (green) and Myh (red) (2), Ucp1 (green) and Myh (red), (3) or GFP (green) and Myh (red) (4). ORO fluorescence is depicted in white. Nuclei were stained with DAPI (blue). Arrows indicate that ORO-positive adipocytes express Ucp1 (3) and nGFP (4). **b**–**d** Significance was calculated by two-way ANOVA test. **b** Quantification of ORO-positive adipocytes that originate from nGFP-expressing satellite cells per cm^2^. **c** Measurement of ORO fluorescence intensity in nGFP-positive satellite cells displayed as percentage of Ctrl. **d** qRT-PCR analysis showing relative transcript levels of indicated genes. **e**–**h** Analyses of FACS sorted control (Ctrl-mCh) and LSD1 overexpressing (LSD1-mCh) satellite cells differentiated for 7 days in adipogenic medium. **e** ORO staining (1) and immunofluorescence assays using antibodies directed against: GFP (green), mCherry (mCh) (red), and Myh (white) (2), Ucp1 (green), mCh (red), and ORO (white) (3), or GFP (green), mCh (red), and ORO (white) (4). Nuclei were stained with DAPI (blue). Arrows indicate that ORO-positive adipocytes express Ucp1 (3) and nGFP (4). **f**–**h** Significance was calculated by two-tailed Student’s *t*-test. **f** Quantification of ORO-positive adipocytes that originate from nGFP-expressing satellite cells per cm^2^. **g** Measurement of ORO fluorescence intensity in nGFP-positive satellite cells displayed as percentage of Ctrl-mCh. **h** qRT-PCR analysis showing relative transcript levels of indicated genes. (**b**, **c**, **f**,**  g**: *n *= 6, **d**, **h**: *n* = 4; mean + SEM, *p < 0.05, **p < 0.01, ***p < 0.001; scale bars: **a** and **e**: (1) 50 µm; (2–4) 100 µm)

To recapitulate our findings in cell culture, we FACS sorted satellite cells^33^ (Supplementary Fig. 4a) from Lsd1^iKO^ mice and induced Lsd1 ablation by Tam treatment in vitro. Alternatively, FACS-sorted satellite cells were isolated from wild-type mice and treated with Lsd1 inhibitor [Lsd1(i)] or vehicle in vitro. Cells were differentiated for 7 days in adipogenic medium. Under these conditions, control population (Ctrl) was composed of a majority of Myh-positive muscle fibers and a small number of Ucp1-expressing brown adipocytes (Fig. 4a, b), in accordance with previous studies^14,32^. In both Lsd1^iKO^ and Lsd1(i)-treated cells, we observed impaired myogenesis (Fig. 4a) with decreased mRNA expression of myogenic markers such as *Myog, Mef2c, Ckm, Myh3*, and *Myl1* (Fig. 4d). Concomitantly, we observed an increased number of ORO-positive adipocytes originating from nGFP-expressing Pax7-positive satellite cells (Fig. 4a, b). In accordance, ORO fluorescence intensity in nGFP-positive cells was increased upon Lsd1 ablation or inhibition (Fig. 4c). It is of note that the observed fat cells were brown adipocytes since they all expressed Ucp1 (Fig. 4a). These data were corroborated by increased transcript levels of the general adipogenic markers *Cebpα, Fabp4*, and *Adipoq*, as well as brown fat selective genes *Pparγ, Prdm16, Ucp1, Elovl3*, and *Cidea* (Fig. 4d). Moreover, western blot analysis showed decreased Myh and elevated Ucp1 protein levels in Lsd1^iKO^ and Lsd1(i) satellite cells compared to Ctrl (Supplementary Figs. 4b and 9c).

To investigate the effect of increased Lsd1 expression levels on adipogenesis, we next infected FACS-sorted satellite cells with an adenovirus containing LSD1 and mCherry (mCh) constructs and differentiated the cells for 7 days in adipogenic medium (Supplementary Fig. 4c). In LSD1 overexpressing cells (LSD1-mCh), we observed increased muscle differentiation to the detriment of brown adipogenesis (Fig. 4e). Decreased fat accumulation was evaluated by counting the number of ORO-positive cells within the nGFP-positive population (Fig. 4f) and corroborated with the ORO fluorescence intensity quantification (Fig. 4g). In addition, LSD1 overexpression led to upregulation of muscle-specific markers and decreased expression of general adipogenic and brown fat selective markers (Fig. 4h). Corresponding phenotypes were obtained in C2C12 cells upon Lsd1 knockdown [Lsd1(KD)] or LSD1 overexpression by stable lentiviral infection [LSD1(OE)] (Supplementary Figs. 4d, e, 5a–f, and 9d, e). In summary, Lsd1 ablation or inhibition in satellite and C2C12 cells leads to increased brown adipocyte differentiation, whereas LSD1 overexpression favors the myogenic phenotype.

### Lsd1 targets muscle-specific genes under myogenic conditions

**Fig. 5:**
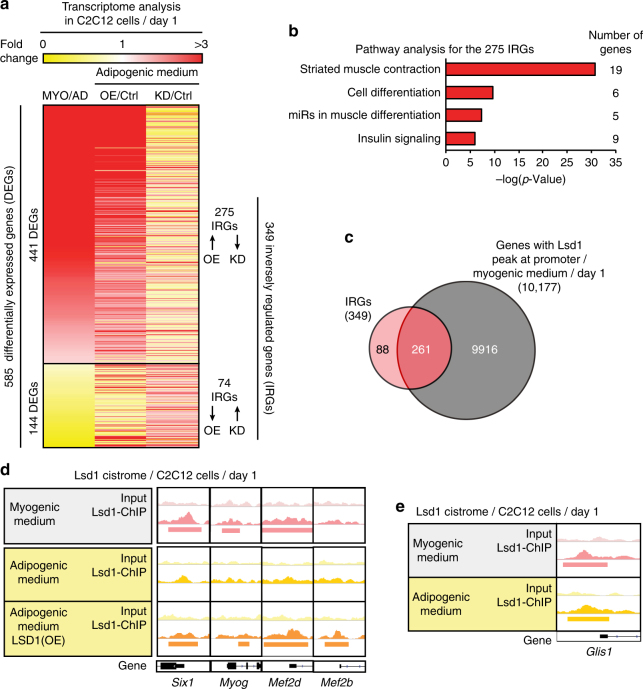
Lsd1 targets muscle-specific genes under myogenic but not under adipogenic conditions. **a** Heatmap obtained from RNA-seq analyses depicting differentially expressed genes (DEGs) in: wild-type C2C12 cells differentiated for 1 day in myogenic versus adipogenic medium (MYO/AD, first column), LSD1 overexpressing versus control C2C12 cells (OE/Ctrl, second column), or Lsd1 knockdown versus control C2C12 cells (KD/Ctrl, third column) differentiated for 1 day in adipogenic medium. **b** Pathway analysis showing significantly enriched networks for the set of 275 inversely regulated genes (IRGs). **c** Overlap of all 349 IRGs (pink) obtained from RNA-seq analyses and genes with Lsd1 promoter occupancy (dark gray) obtained from ChIP-seq analyses in C2C12 cells differentiated for 1 day in myogenic medium. **d** ChIP-seq tracks depicting Lsd1 chromatin occupancy at indicated promoters in wild-type C2C12 cells differentiated for 1 day in myogenic or adipogenic medium or in LSD1 overexpressing [LSD1(OE)] C2C12 cells differentiated for 1 day in adipogenic medium. Identified peaks are marked with bars. **e** ChIP-seq tracks depicting Lsd1 chromatin occupancy at the promoter of *Glis1* in C2C12 cells differentiated for 1 day in myogenic or adipogenic medium. Identified peaks are marked with bars. (**a**, **b**: *n* = 3)

Next, we aimed to uncover the molecular mechanism by which Lsd1 drives differentiation and fate decision of muscle precursors. First, to identify genes whose transcript levels change at the onset of myogenic versus adipogenic differentiation of myoblasts, we compared the transcriptomes of wild-type C2C12 cells differentiated for 1 day in either myogenic or adipogenic medium. Numbers of differentially expressed genes (DEGs) are depicted in Supplementary Fig. 6a. Next, to identify genes whose expression is affected by altered Lsd1 levels, we performed RNA-seq analyses in C2C12 cells upon LSD1 overexpression or Lsd1 knockdown in adipogenic medium and compared them to corresponding control cells (Supplementary Fig. 6b). Since under these conditions we observed predominantly adipogenic or myogenic phenotype, respectively, we reasoned that by overlapping all three data sets, we might find genes responsible for fate specification between muscle and fat. The overlap revealed a total of 585 common DEGs, from which 441 were upregulated and 144 were downregulated in myogenic versus adipogenic differentiation (Fig. 5a). Upon LSD1 overexpression in adipogenic medium, the vast majority of 585 DEGs showed a similar expression pattern as during myogenic differentiation (Fig. 5a), On the contrary, upon Lsd1 knockdown in adipogenic medium, the majority of the 585 DEGs showed a mirror image of expression (Fig. 5a), indicating that Lsd1 drives the transcriptional program that discriminates myogenic from adipogenic differentiation. We hypothesized that potential Lsd1 target genes driving C2C12 cell commitment should be inversely regulated upon Lsd1 knockdown and overexpression. From 349 inversely regulated genes (IRGs), 275 were upregulated in LSD1(OE) and downregulated in Lsd1(KD) cells compared to control (Supplementary Table 1). For this gene group ‘*striated muscle contraction*’ pathway was strongly enriched (Fig. 5b), containing numerous muscle structural genes, as well as genes encoding muscle transcription factors. For the remaining 74 IRGs that were downregulated in LSD1(OE) and upregulated in Lsd1(KD) cells compared to control (Supplementary Table 2), none of the previously described pathways were significantly enriched.

To identify direct Lsd1 target genes involved in muscle specification and function, we performed ChIP-seq analysis in C2C12 cells differentiated for 1 day in myogenic medium using a previously validated Lsd1 antibody^24,28,34^ (Supplementary Fig. 6c). We found that the majority of the 349 IRGs were direct Lsd1 targets, as 261 (75%) of them displayed Lsd1 promoter occupancy (Fig. 5c). In myogenic medium, Lsd1 was present at the promoters of key muscle transcription factor genes such as *Six1*, *Myog*, and *Mef2d* (Fig. 5d), as well as structural muscle genes such as *Mb, Myh1, Myh3, Myl1, Ttn, Tnnt3*, and *Tnnc1* (Supplementary Fig. 6d). These results are in accordance with published data^22^, confirming that Lsd1 activates the expression of myogenic genes. Since we were interested in fate specification of myoblasts, we investigated Lsd1 occupancy of muscle-specific promoters in wild-type and LSD1-overexpressing C2C12 cells differentiated for 1 day in adipogenic medium (Supplementary Fig. 6c). ChIP-seq results showed that in adipogenic medium, only 181 out of 349 IRGs (52%) were direct Lsd1 targets (Supplementary Fig. 6e). Under adipogenic conditions Lsd1 was not present on muscle genes (Fig. 5d and Supplementary Fig. 6d), but upon overexpression it was found at the promoters of *Six1, Myog, Mef2d*, and *Mef2b* (Fig. 5d). These data are in full agreement with our findings that in LSD1(OE) C2C12 cells myogenesis is favored to the detriment of brown adipogenesis. Together, RNA-seq and ChIP-seq data showed that Lsd1 directly regulates the myogenic gene program in C2C12 cells in myogenic medium, explaining how elevated Lsd1 levels in transgenic mice enhance muscle differentiation upon injury.

### Lsd1 represses expression of the pro-adipogenic factor Glis1

Next, we wanted to uncover the mechanism by which Lsd1 prevents adipogenic differentiation of myoblast precursors during muscle regeneration. We hypothesized that this might be accomplished either by upregulation of adipogenic repressor genes or by repression of pro-adipogenic genes. However, among the 349 IRGs we did not identify any known pro- or anti-adipogenic factor. Searching for a novel adipogenic regulator, we noticed that the proline-rich Krüppel-like transcription factor Glis1 was strongly upregulated upon Lsd1 knockdown and downregulated upon LSD1 overexpression in C2C12 cells (Supplementary Table 2). These observations suggested that Glis1 acts as a novel pro-adipogenic factor repressed by Lsd1 during physiological myogenic differentiation.

**Fig. 6:**
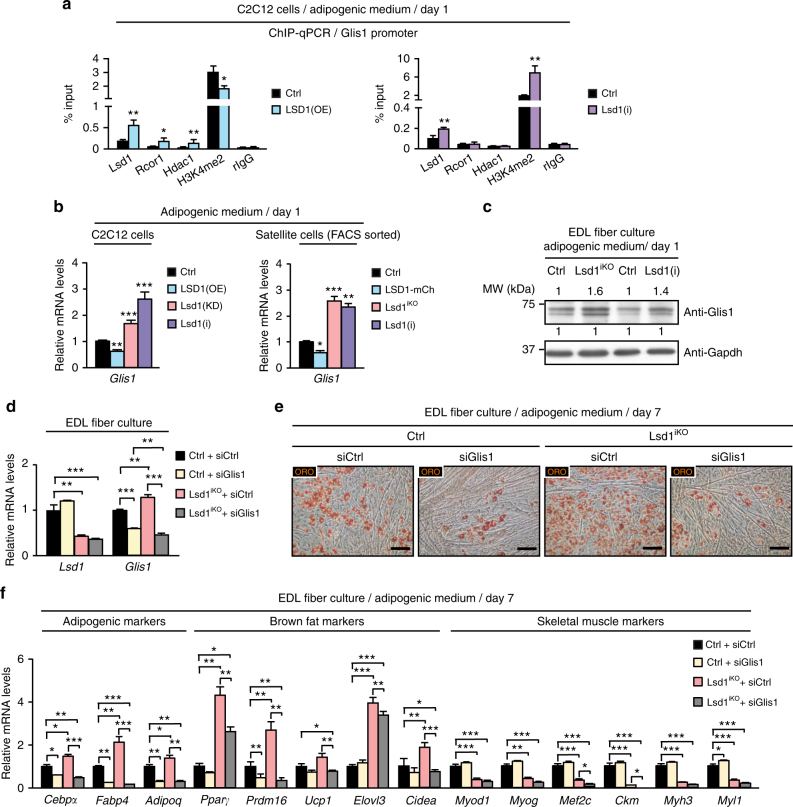
Lsd1 represses expression of the pro-adipogenic factor Glis1. **a** ChIP-qPCR analyses using antibodies directed against Lsd1, REST corepressor 1 (Rcor1), histone deacetylase 1 (Hdac1), dimethylated histone 3 lysine 4 (H3K4me2), or rabbit immunoglobulin G (rIgG) in control (Ctrl) and LSD1(OE) C2C12 cells (left panel), or Lsd1 inhibitor-treated [Lsd1(i)] C2C12 cells (right panel) differentiated for 1 day in adipogenic medium. Immunoprecipitated chromatin was quantified by qPCR using primers flanking Lsd1-binding sites at the promoter of *Glis1* gene. **b** qRT-PCR analyses showing relative transcript levels of *Glis1* in Ctrl, LSD1(OE), Lsd1 knockdown [Lsd1(KD)], and Lsd1(i) C2C12 cells (left panel), or Ctrl, LSD1-GFP, Lsd1^iKO^, and Lsd1(i) satellite cells (right panel) differentiated for 1 day in adipogenic medium. Significance was calculated by two-tailed Student’s *t*-test. **c** Western blot analysis showing protein levels of Glis1 in Ctrl, Lsd1^iKO^, and Lsd1(i) satellite cells differentiated for 1 day in adipogenic medium. **d**–**f** Analyses of Ctrl and Lsd1^iKO^ EDL fiber culture cells transfected with control (siCtrl) or siRNA directed against *Glis1* (siGlis1) and differentiated for 7 days in adipogenic medium. **d** qRT-PCR analyses showing relative transcript levels of *Glis1* and *Lsd1*, **e** ORO staining, and **f** qRT-PCR analyses showing relative transcript levels of indicated genes. (**a**,** d**,** f**: *n* = 3, **b**: *n* = 6; mean + SEM, *p < 0.05, **p < 0.01, ***p < 0.001; scale bars: **e**: 50 µm)

Inspection of our cistrome data uncovered that Lsd1 occupies the Glis1 promoter in C2C12 cells under adipogenic and myogenic conditions (Fig. 5e). To corroborate the genome-wide data, we verified Lsd1 occupancy of the *Glis1* promoter by performing ChIP-qPCR analysis in LSD1(OE) and Ctrl C2C12 cells differentiated for 1 day in adipogenic medium. Lsd1 was present at the *Glis1* promoter and binding was significantly increased in LSD1(OE) C2C12 cells compared to Ctrl (Fig. 6a). Concomitant with increased Lsd1 binding in LSD1(OE) cells, we detected recruitment of histone deacetylase 1 (Hdac1) and REST corepressor (Rcor1), members of the CoREST repressive complex (Fig. 6a). Accordingly, H3K4me2 levels were significantly decreased in LSD1(OE) C2C12 cells and, on the contrary, increased in Lsd1(i) C2C12 cells (Fig. 6a). Despite increased Lsd1 binding at the Glis1 promoter upon inhibitor treatment, binding of other complex members such as Hdac1 and Rcor1 was not detected (Fig. 6a). Increased H3K4me2 levels at *Glis1* promoter were also observed in Lsd1^iKO^ and Lsd1(i)-treated satellite cells (Supplementary Fig. 7a). These results suggest that repressive action of Lsd1 is mediated by the removal of active H3K4me2 mark. In agreement, transcript levels of *Glis1* were decreased upon LSD1 overexpression and increased upon Lsd1 ablation or inhibition in C2C12 and satellite cells at day 1 of adipogenic differentiation (Fig. 6b). This observation is supported by western blot analysis showing increased Glis1 protein levels in Lsd1^iKO^ and Lsd1(i) satellite cells compared to Ctrl (Fig. 6c and Supplementary Fig. 9f). These data show that Lsd1 directly represses the expression of Glis1 in C2C12 and satellite cells, and that Lsd1 ablation or inhibition leads to upregulation of Glis1 at both mRNA and protein level.

To test whether Glis1 plays a role in adipogenesis of satellite cells, we treated satellite cells derived from EDL fiber culture with control siRNA (Ctrl + siCtrl) or siRNA directed against *Glis1* (Ctr + siGlis1) at day 1 of adipogenic differentiation (Fig. 6d). After 7 days, reduced Glis1 expression resulted in impaired fat accumulation (Fig. 6e) and decreased expression of adipogenic markers compared to siCtrl-treated cells suggesting that indeed Glis1 has pro-adipogenic activity (Fig. 6f). Of note, expression of muscle-specific genes was not affected by Glis1 knockdown (Fig. 6f). To assess whether Glis1 mediates the adipogenic phenotype observed upon Lsd1 ablation, we knocked down Glis1 in Lsd1^iKO^ satellite cells (Fig. 6d). Importantly, reduced Glis1 levels in Lsd1^iKO^ cells (Lsd1^iKO^ + siGlis1) robustly antagonized excessive lipid accumulation observed in siCtrl-treated Lsd1^iKO^ cells (Lsd1^iKO^ + siCtrl) and strongly diminished the expression of adipogenic markers (Fig. 6e, f). Of note, downregulation of muscle-specific genes expression caused by Lsd1 ablation was not restored by Glis1 knockdown (Fig. 6f). Similar results were obtained when Glis1 was knocked down in satellite cells treated with Lsd1 inhibitor (Supplementary Fig. 7b, c). In summary, our data show that the Lsd1 target gene Glis1, at least in part, promotes the adipogenic fate of satellite cells without affecting muscle-specific genes.

### GLIS1 overexpression favors brown adipocyte differentiation

Having identified Glis1 as a pro-adipogenic factor, we first evaluated physiological Glis1 protein levels during myogenic and adipogenic differentiation of satellite and C2C12 cells. Glis1 was expressed at high levels in undifferentiated cells (Supplementary Figs. 8a, b and 9g), which is in accordance with its published role in maintaining pluripotency^35^. Under myogenic conditions, Glis1 levels were drastically decreased from day 1 to 7 of differentiation (Supplementary Fig. 8a, b). In adipogenic medium, peak of Glis1 expression was reached at day 1 of differentiation, followed by a strong decline from day 4 to 7 (Supplementary Fig. 8a, b). Of note, Glis1 levels were higher in adipogenic compared to myogenic medium, corroborating its role in adipogenesis.

**Fig. 7:**
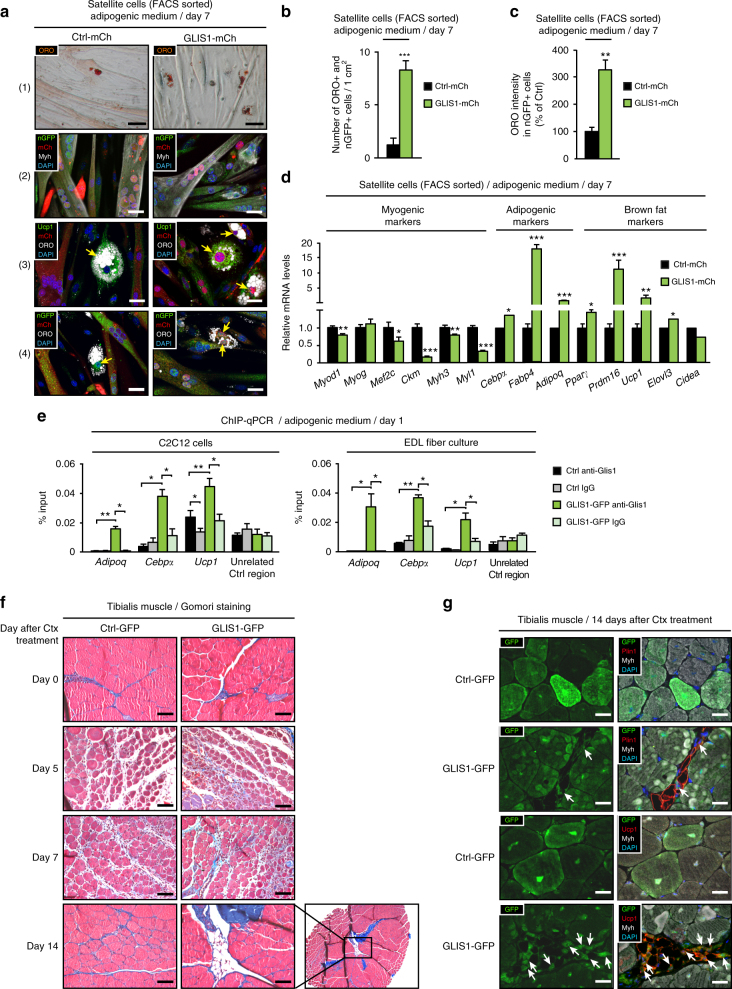
GLIS1 overexpression favors brown adipocyte differentiation of satellite cells. **a**–**d** Control (Ctrl-mCh) and GLIS1 overexpressing (GLIS1-mCh) FACS-sorted satellite cells differentiated for 7 days in adipogenic medium. **a** ORO staining (1) and immunofluorescence assays using antibodies directed against: GFP (green), mCh (red), and Myh (white) (2), Ucp1 (green), mCh (red), and ORO (white) (3), or GFP (green), mCh (red), and ORO (white) (4). Nuclei were stained with DAPI (blue). Arrows indicate that GLIS1-mCh adipocytes express Ucp1 (3) and nGFP (4). **b**–**d** Significance was calculated by two-tailed Student’s *t*-test. **b** Quantification of ORO-positive adipocytes that originate from nGFP-expressing satellite cells per cm^2^. **c** Measurement of ORO fluorescence intensity in nGFP-positive satellite cells displayed as percentage of Ctrl. **d** qRT-PCR analysis showing relative transcript levels of indicated genes. **e** ChIP-qPCR analyses using Glis1 antibody or rIgG control on Ctrl-GFP or GLIS1-GFP C2C12 and satellite cells differentiated for 1 day in adipogenic medium. Immunoprecipitated chromatin was quantified by qPCR analysis using primers for the promoter regions of *Cebpα*, *Adipoq*, and *Ucp1*, or an unrelated control region. **f**, **g** Representative tibialis muscle sections from wild-type muscle injected with Ctrl-GFP or GLIS1-GFP adenovirus 0, 5, 7, and 14 days after Ctx treatment. **f** Gomori staining, and **g** immunofluorescence assays using antibodies directed against GFP (green), Plin1 (red), and Myh (white) or GFP (green), Ucp1 (red), and Myh (white) as indicated. Nuclei were stained with DAPI (blue). Arrows indicate that adipocytes express GFP (**b**,** c**: *n* = 6, **d**,** e**: *n* = 4; mean + SEM, *p < 0.05, **p < 0.01, ***p < 0.001; scale bars: **a** (1), **g** 50 µm, **a **(2-4), **f** 100 µm)

Next, we tested whether GLIS1 overexpression is sufficient to drive cell fate of satellite cells towards brown fat differentiation and therefore mimic the effects of Lsd1 ablation. For this purpose, we overexpressed GLIS1 protein in FACS-sorted satellite cells by infection with GLIS1-mCh adenovirus (Supplementary Fig. 8c) and differentiated them in adipogenic medium for 7 days. Of note, GLIS1 overexpression did not affect transcript expression of *Lsd1* (Supplementary Fig. 8d). GLIS1 overexpressing cells were characterized by increased adipogenesis compared to control cells (Ctrl-mCh), as demonstrated by an increased number of ORO-, mCh-, and nGFP-positive cells (Fig. 7a, b) and increased ORO fluorescence intensity (Fig. 7c). Additionally, we observed increased mRNA levels of general adipogenic and brown fat selective markers, as well as a downregulation of muscle markers (Fig. 7d). Of note, muscle-specific transcriptional repertoire was decreased to a less extent compared to Lsd1-ablated cells. Together, these results confirmed that Glis1 acts as a pro-adipogenic transcription factor in satellite cells, and that its overexpression is sufficient to enhance brown adipogenesis.

To determine whether Glis1 directly regulates adipogenic genes to drive brown adipogenesis of muscle precursors, we overexpressed GLIS1 in C2C12 and satellite cells derived from EDL fiber culture (Supplementary Figs. 8e and 9h) and performed ChIP-qPCR experiments using primers encompassing Glis1-binding motifs at promoters of adipogenic genes. ChIP-qPCR experiments showed increased Glis1 occupancy of the *Adipoq, Cebpa*, and *Ucp1* promoters in GLIS overexpressing (GLIS1-GFP) C2C12 and satellite cells compared to control (Ctrl-GFP) (Fig. 7e). Of note, no Glis1 binding was detected at unrelated control region (Fig. 7e). In summary, our data show that GLIS1 overexpression enhances brown adipogenesis of satellite cells. Furthermore, in GLIS1-GFP C2C12 and satellite cells Glis1 is present at the promoters of adipogenic genes such as *Adipoq, Cebpα*, and *Ucp1*, providing a molecular mechanism by which Glis1 drives satellite cell fate towards brown adipogenesis.

Finally, to investigate whether GLIS1 overexpression would lead to brown fat differentiation during muscle regeneration in vivo, we injected GLIS1-GFP or Ctrl-GFP adenovirus into the tibialis muscle of wild-type mice 1 day after Ctx-induced injury and allowed muscle to regenerate for 0, 5, 7, or 14 days. Injection of GLIS1-GFP resulted in the appearance of Plin1-positive and Ucp1-positive brown adipocytes within muscle fibers (Fig. 7f, g and Supplementary Fig. 8f). Brown fat accumulation was neither observed after the injection of Ctrl-GFP adenovirus into the Ctx-treated muscle, nor upon injection of GLIS1-GFP or Ctrl-GFP adenoviruses into untreated muscle (Fig. 7f, g). Increased GLIS1 levels did not seem to dramatically affect muscle regeneration, since quantification of regenerating fibers in Ctrl-GFP and GLIS1-GFP muscle 5 and 7 days after Ctx injection did not show significant differences (Supplementary Fig. 8g). At day 5 post Ctx injury, GLIS1 overexpressing muscle had higher number of fibers with smaller area compared to control, but this difference is no longer observable at day 7 after Ctx injection (Supplementary Fig. 8h). Our data show that GLIS1 overexpression during muscle regeneration in vivo is sufficient to mimic the phenotype of Lsd1 ablation or inhibition and cause brown fat infiltration in regenerating muscle with minor effect on muscle differentiation.

In conclusion, Lsd1 regulates myogenic differentiation of satellite cells during muscle regeneration and restricts their fate by inhibiting brown adipogenesis. Lsd1 accomplishes this action by repressing the expression of a novel pro-adipogenic factor Glis1. Lsd1 ablation or inhibition causes increased Glis1 expression and transcriptional activation of general adipogenic and brown fat-specific genes, leading to a fate switch of satellite cells and their differentiation into brown adipocytes. Maintaining physiological levels of Lsd1 is therefore necessary for proper muscle recovery upon injury.

## Discussion

In the present study, we identified Lsd1 as a key epigenetic regulator of skeletal muscle regeneration, since elevated Lsd1 levels accelerate muscle recovery upon injury, while Lsd1 ablation or inhibition directs satellite cell fate towards brown adipocytes. The role of Lsd1 in muscle differentiation has been implicated by several studies proposing that Lsd1 interacts with Myod1 and Mef2 proteins^20,22^ to demethylate H3K9me2 and activate the expression of *Myog* and *Ckm* genes^22^. Our cistrome data are in accordance with published transcript analyses and provide additional insights into the regulation of muscle-specific genes by Lsd1.

More importantly, however, our data provide first insights into the role of epigenetic erasers in muscle regeneration. Lsd1 ablation or inhibition not only delays skeletal muscle regeneration but switches the fate of satellite cells towards brown adipocytes. Under physiological conditions, Lsd1 restricts the adipogenic potential of satellite cells via repression of the transcription factor Glis1. Glis1 was previously reported to increase the reprogramming efficiency of somatic cells by increasing pluripotency^36^. Here, we describe a novel function of Glis1 in regulating adipogenic genes in satellite cells. Our data indicate that this mechanism is functional in vivo, since GLIS1 overexpression in murine regenerating muscle is sufficient to induce the appearance of brown adipocytes within muscle fibers.

Several studies implicated Lsd1 in the differentiation of white and beige adipocytes. Musri et al.^23^ showed that Lsd1 knockdown markedly decreases adipogenic differentiation of 3T3-L1 and C3H-10T1/2 pre-adipocytes. These findings are recapitulated in vivo, since Lsd1 knockout using Fatty acid binding protein 4 (Fabp4)-Cre deleter strain led to complete absence of visceral and subcutaneous fat in mice^24^. Consistently, it was demonstrated that Lsd1 promotes brown adipogenesis^25^ since newborn mice in which Lsd1 was ablated in Myf5-positive precursors were devoid of BAT^37^. However, our current study shows that during muscle regeneration, Lsd1 restricts brown adipocyte fate of satellite cells and promotes myogenesis. Thus, in muscle and adipose tissues Lsd1 drives the cell fate in opposite directions. Diverse Lsd1 functions are most probably achieved by targeting and/or interacting with tissue-specific transcription factors. For example, it was showed that in myoblasts Lsd1 interacts with myogenic factors Myod1 and Mef2d, which cooperate in the regulation of the expression of muscle-specific genes^22^.

In line with these diverse roles in muscle and adipose stem cells is the function of Lsd1 in the regulation of thermogenic properties of mature adipocytes. In white adipose tissue Lsd1 is necessary to maintain oxidative and thermogenic capacities of beige adipocytes^24^. We recently demonstrated that age-dependent decline of Lsd1 is responsible for the conversion of beige adipocytes into white inguinal and epididymal WAT during aging^38^. Similarly, ablation or enzymatic inhibition of Lsd1 in brown adipocytes leads to metabolic reprogramming and induces whitening of BAT^28^. In both cases, loss of Lsd1 led to downregulation of Ucp1 protein. In this study, we show that brown adipocytes emerging from satellite cells upon Lsd1 ablation express Ucp1 protein (at lower levels compared to brown adipocytes), as well as a repertoire of thermogenic/oxidative markers. Altogether, our data show that cell-type-specific transcriptional repertoires are most probably governed via the action of different Lsd1-containing complexes and/or target genes.

Taken together our results implicate that physiological levels of Lsd1 are necessary for normal muscle regeneration after injury. Elevated levels of Lsd1 might be beneficial and accelerate the process of muscle regeneration, whereas loss or inhibition of Lsd1 leads to aberrant differentiation and acquisition of a brown fat phenotype. Lsd1 therefore, not only promotes muscle differentiation, but also restricts the fate of satellite cells to the myogenic lineage. Understanding the molecular underpinnings by which Lsd1 regulates the myogenic-adipogenic switch might open novel therapeutic perspectives for muscle regeneration from injury caused by trauma or degeneration in muscle dystrophies and aging.

## Methods

### Cell culture

Satellite cells were either obtained by FACS sorting or isolated from EDL fiber culture as indicated^32,33^. Cells were proliferated in DMEM high glucose (4.5 g/l) and sodium pyruvate (110 mg/l) medium (Thermofisher Scientific, 11995-065) supplemented with 20% FCS, 1% penicillin/streptomycin, and 1% chicken embryo extract for ~4 days. When cells were 50–60% confluent, they were infected with LSD1, GLIS1, or control adenovirus (Vector BioLabs) with multiplicity of infection of 200. Two days after infection, cells were differentiated in myogenic medium (DMEM high glucose and sodium pyruvate supplemented with 2% horse serum and 1% penicillin/streptomycin) or adipogenic medium (DMEM high glucose and sodium pyruvate supplemented with 10% FBS, 0.5 mM isobutylmethylxanthine, 125 nM indomethacin, 5 μM dexamethasone, 850 nM insulin, 1 nM T3, and 1 μM rosiglitazone). Two days after inducing adipogenic differentiation, cells were switched to the maintenance medium containing 10% FBS, 850 nM insulin, 1 nM T3, and 1 μM rosiglitazone. Cells were harvested and analyzed 7 days after induction of differentiation. To obtain Lsd1-ablated cells, satellite cells were isolated from Lsd1^iKO^ mice and in vitro treated with 1 μM 4-hydroxitamoxifen 5 days before induction of differentiation.

C2C12 cells were obtained from ATCC (CRL-1772). Stable inducible LSD1 overexpressing C2C12 cell line was generated by infecting the cells with the lentivirus containing pSlik-Neo tetracycline-controlled transactivator (tTA) plasmid with Flag-tagged LSD1 construct downstream of the tetracycline-dependent promoter and selecting with G418 (1 mg/ml). To induce LSD1 overexpression, cells were treated with doxycycline (2 µg/ml) 48 h prior to differentiation and throughout the differentiation process. Cells were grown in DMEM high glucose medium and myogenic or adipogenic differentiation were induced as described for satellite cells. Lsd1 or Glis1 knockdown were induced by transfecting the cells with 20 nM siRNA against Lsd1 or Glis1, respectively (Invitrogen) using DharmaFECT 3 (ThermoFicher Scientific) according to the manufacturer’s instructions. siRNA oligonucleotide sequences used were as follows:

Lsd1 siRNA: 5′-CCC AAA GAU CCA GCU GAC GUU UGA A-3′

Unrelated control siRNA: 5′-CCC UAG AGA CCA GUC UUG CUA AGA A-3′

Glis1 siRNA: 5′-CCU CGA CAC GAA GCC AUA UGC UUG U-3′

Unrelated control siRNA: 5′-GUC CCG ACC AAC UGU GGCU UAA CUA-3′

To inhibit enzymatic activity of Lsd1 in satellite cells and C2C12 cells, we used specific irreversible nanomolar affinity Lsd1 inhibitor ORY-1001 (Oryzon)^30^. Cells were treated with 50 nm inhibitor 2 days prior to differentiation and throughout 7 days of differentiation. Equivalent volume of DMSO was used as a vehicle. Differentiated satellite and C2C12 cells were either harvested and snap-frozen in liquid nitrogen to be used for RNA and protein extraction, or fixed on two-well matrigel-coated slides (Ibidi, 80286) to be used for immunofluorescence assay.

### FACS sorting of satellite cells

FACS sorting of satellite cells was performed as published^33^. In brief, to isolate satellite cells, we dissected hind limb and trunk muscles from wild-type, Lsd1^iKO^, and Lsd1^iKI^ mice, minced them into small pieces, digested with 2.2 U/ml Dispase II (Sigma Aldrich, D4693) and 0.6% type II collagenase (Worthington Biochemicals, LS004176), and filtered through 100 μm, 70 μm, and 40 μm cell strainers (Corning Life Sciences). After lysis of red blood cells by hypotonic shock (150 mM NH_4_Cl, 100 mM KHCO_3_, 125 mM EDTA, pH 7.35) cell suspension was loaded onto a 30%/70% Percoll (Sigma-Aldrich, P-4937) gradient. Mononuclear cells we collected from the 30% and 70% Percoll interphase, and immunofluorescence assay was performed using fluorescence-coupled primary antibodies directed against: CD11b PE-Cy7 (eBioscience, 25-0112-82, 1:100), CD45 PE (eBioscience, 12-0451-83, 1:100), CD31 PE (eBioscience, 12-0311-82, 1:100), Integrin-α7 FITC (MBL, K0046-4, 1:100), CXCR4 APC (eBioscience, 17-9991-82, 1:20), and CD34 eFluor450 (eBioscience, 48-0341-82, 1:33). Cells were gated based on the forward scatter (FSC) and side scatter (SSC) parameters to exclude cellular debris and cell doublets. Next, the CD11b, CD45, and CD31 markers were used to exclude leukocytes and endothelial cells. CD11b/CD45/CD31-negative cells were further selected for the expression of CD34 and Integrin-α7 markers. Finally, double positive cells were gated for the expression of CXCR4. Sorted cells were cultured on matrigel-coated eight-well slides (Ibidi, 80826).

### Immunocytochemistry

Immunocytochemistry assay was performed by incubating fixed C2C12 or satellite cells for 15 min in permeabilization buffer (PBS with 0.2% Triton-X 100 and 0.1% Tween 20) followed by 1 h fixation in PBS with 0.1% Tween 20 and 5% FBS (Gibco, 10270-106). Cells were stained over night at 4 °C using the following antibodies: pan-myosin heavy chain (Myh_fast_, Sigma, M4276, 1:1000 and Myh_slow_, Sigma, M8421, 1:1000), Lsd1 (Custom made, Sigma, 112-4, 1:500), or GFP (Abcam, ab13970, 1:500). Secondary antibodies were conjugated with AlexaFlour-488 and AlexaFlour-547 fluorescent dyes (ThermoFisher Scientific, A-11034 and A-11029 respectively, 1:400) and incubated on the cells for 1 h at room temperature. Nuclei were visualized by DAPI stain (Sigma, D-9542, 1:10000). Stained cells were observed under Leica confocal microscope.

### Immunohistochemistry

For immunohistochemistry, tissues were fixed in 10 % buffered formalin and embedded in paraffin. 5 μm paraffin sections were deparaffinized and rehydrated, and antigen retrieval was performed by boiling the samples for 20 min in pH 9 Tris buffer. Sections were blocked for 1 h (PBS with 0.1% Tween 20 and 5% FBS) and incubated overnight at 4 °C with primary antibodies directed against: Lsd1 (Cell signaling, 2184, 1:400), pan-Myosin heavy chain (Myh_fast_, Sigma, M4276, 1:1000 and Myh_slow_, Sigma, M8421, 1:1000), Dmd (Abcam, ab15277, 1:500), Pax7 (Custom-made, DSHB, 1:80), GFP (Abcam, ab13970, 1:500), Plin1 (Abcam, ab3526, 1:400), or Ucp1(Abcam, ab10983, 1:500). Slides were incubated with appropriate secondary antibodies conjugated with AlexaFlour-488, AlexaFlour-547, or AlexaFluor-633 fluorescent dyes (ThermoFisher Scientific, A-11034, A-11029 and A-21050 respectively, 1:400). Nuclei were visualized by DAPI stain. Slides were mounted in aqueous medium (Fluoromount-G, SouthernBiotech, 0100-01) and observed using Leica confocal microscope.

### Gomori trichrome staining

Gomori trichrome staining was performed using a Trichrome Stain Kit according to the manufacturer recommendations with modifications (ScyTek Laboratories). Samples were deparaffinized, rehydrated, and incubated for 1 h in Bouin’s solution preheated to 65 °C under a fume hood. Slides were incubated 10 min in Weigert’s iron hematoxylin stain at room temperature. Excess stain was removed by rinsing in water and acid alcohol solution. Samples were next stained with Trichrome stain solution for 20 min at room temperature, followed by washing in water and acetic acid solution. Slides were dehydrated in alcohol and xylol, mounted with RotiHisto Kit mounting medium (Carl Roth, 66381), and observed under Zeiss Axioskop 2 microscope.

### Oil Red O staining

Oil red O (ORO) staining was performed as described^39^. Fixed cells were incubated in ORO dye solution for 30 min. Staining was observed under a phase-contrast microscope or in the far-red spectrum under Leica confocal microscope.

### Animals

All mice were housed in the pathogen-free barrier facility of the University Medical Center Freiburg in accordance with institutional guidelines and approved by the regional board. Mice were maintained in a temperature- and humidity-controlled animal facility with a 12-h light/dark cycle, free access to water, and a standard rodent chow (Kliba, breeding, 3807). All experiments were performed on C57/Bl6N mouse strain at 9–11 weeks of age. Primers used for genotyping of the mouse tissue are listed in Supplementary Table 3.

### Generation of conditional LSD1 overexpressing mice

Generation of the targeting vector was described previously^38^. Mice carrying pβ-actin-att-IRES-lo_LSD1 vector were crossed to the well described Myf5-Cre deleter strain^31^ to induce LSD1 overexpression in Myf5-positive precursors.

### Generation of conditional Lsd1 knock-out and knock-in mice

The targeting strategy for the conditional deletion of the first exon of Lsd1 (Kdm1a^tm1.1Rosc^)^27^ and conditional *Lsd1* mutant knock-in alleles^28^ was described previously. These mice were crossed with mice expressing tamoxifen (Tam) inducible *Cre recombinase* under the control of the *Pax7* promoter (Pax7-CreERT2)^26^ to selectively ablate Lsd1 in satellite cells. These mice also express Rosa26-GNZ knock-in allele^29^ enabling expression of a nuclear-localized GFP and beta-galactosidase fusion protein (GFP-NLS-*lacZ* or GNZ) once an upstream *loxP*-flanked STOP sequence is recombined. Tam-treated homozygous conditional mice with Rosa26-GNZ knock-in allele were used as controls.

### Muscle regeneration experiments

To induce Lsd1 ablation or inhibition, 10-weeks-old Lsd1^iKO^ and Lsd1^iKI^ mice were injected with Tam intraperitoneally every 24 h for five consecutive days (10 mg/ml at 75 mg/kg). Seven days after Tam treatment, muscle regeneration experiments were performed by injecting cardiotoxin (Ctx). Total of 50 µl of 0.06 mg/ml Ctx from *Naja mossambica* snake (Sigma Aldrich, C9759) was injected into the left hind limb *tibialis* muscle of 10-weeks-old mice. To observe the effect of chemical Lsd1 inhibition on muscle regeneration, wild-type tibialis muscle was injected with 20 µl of Lsd1 inhibitor ORY-1001 (Oryzon, 1.5 μg/μl in 0.5% methyl cellulose) 1 day following Ctx injury. To test the effect of the GLIS1 overexpression on muscle regeneration, tibialis muscle was injected with 30 µl of 4 × 10^9^ plaque formation units (PFU) of GLIS1 adenovirus 1 day after Ctx injury.

### Lipid extraction and quantification

Free fatty acids were extracted with a chloroform-free kit according to the manufacturer recommendations (Cell BioLabs). Total quantity of lipids was measured using the colorimetric sullfo-phospho-vanillin assay (SPVA, Cell BioLabs). Genomic DNA isolated with Roti-Phenol/Chloroform/Isoamyl alcohol (Carl Roth) and precipitated with isopropanol was used to normalize the lipid content to the total number of cells.

### qRT-PCR and RNA sequencing

Total RNA was isolated using TRIzol reagent (Invitrogen, 15596018) and precipitated with isopropanol. RNA was isolated with TRIzol Reagent (Invitrogen). Two micrograms of RNA were converted to cDNA using SuperScript II reverse transcriptase (Invitrogen) and polyT oligonucleotides according to the supplier’s protocol. Quantitative RT-PCR was performed using the Abgene SYBR Green PCR kit (Invitrogen) according to the supplier’s protocol. Data were analyzed using the standard curve method^40^. Hprt, 36b4, or Ppia were used for normalization. Primer sequences used for qRT-PCR analyses are provided in Supplementary Table 4. For RNAseq, RNA was isolated using TRIzol reagent, RNA integrity was confirmed by Bioanalyzer and cDNA library preparation and sequencing was performed (HiSeq 2000, paired-end, 100 bp). RNA samples were sequenced by the standard Illumina protocol to create raw sequence files (.fastq files). Reads were mapped to the mouse mm10 genome (NCBI Build 37) using TopHat version 2 (http://ccb.jhu.edu/software/tophat). The aligned reads were counted with the Homer software (analyzeRepeats.pl)^41^ and differentially regulated genes were identified using EdgeR^42^. As a threshold for DEGs we chose *p* value < 10^−5^, minimum 50 reads, and fold change excluding values between 0.77 and 1.3. Pathway analysis were performed in WebGestalt^43^. Data are available online in the GEO database (GSE98133).

### Protein isolation and western blot analyses

Whole cell extract was obtained from cell pellets by extraction in SC buffer (50 mM Tris-HCl pH 8.0, 170 mM NaCl, 0.1% NP40, Complete EDTA-free protease inhibitor cocktail (Sigma-Aldrich, 11873580001)). Protein isolation from tissue samples was performed with RIPA buffer (1 mM EDTA, 50 mM Tris-HCl pH 7.5, 0.1% SDS, 150 mM NaCl, 1% NP40, 1% Sodium deoxycholate, Complete EDTA-free protease inhibitor cocktail). Tissues were lysed by Minilys personal homogenizer (Bertin Corp.). Western blot analyses were performed as described^24^. Membranes were decorated using antibodies directed against: Lsd1 (Custom-made, Biogenes, 3544, 1:1000), Tubulin (Sigma-Aldrich, T6074, 1:10,000), FlagM2 (Sigma-Aldrich, F3165, 1:2000), Ucp1 (Abcam, ab10983, 1:500), Myh_fast_ (Sigma-Aldrich, M4276, 1:1000), Gapdh (Santa Cruz, sc-47724, 1:5000), or Glis1 (Abcam, ab135724, 1:200). Western blot results were quantified using an Amersham Imager 6000 and Melanie 2D Gel Analysis Software.

### Chromatin immunoprecipitation

For ChIP-seq experiment, C2C12 cells were fixed for 6 min with 1% PFA at 4 °C followed by 5 min blocking in 125 mM glycine. Nuclei preparation was performed using previously described NEXON protocol^44^, by sonicating the cells with Covaris E220 focused ultrasonicator for 2 min at peak power 75 W, duty factor 2% and 200 cycles/burst at 4 °C. Chromatin was sheared using Covaris E220 focused ultrasonicator to obtain a fragment size distribution of 100–800 bp (peak power: 140 W; duty factor: 5%; cycles/burst: 200, water temperature 4 °C). Chromatin was immunopercipitated using homemade antibody directed against C-terminus of Lsd1 protein (Biogenes, 20752) as described^24^. ChIP-seq libraries were prepared, sequenced using the standard Illumina protocol (HiSeq2000, single read, 50 bp v3), and mapped to the mouse mm10 reference genome by Bowtie^45^. Data were further analyzed using the peak finding algorithm MACS 1.4.2^46^. Homer software was used to annotate peaks^47^, and all peaks with false discovery rate <1% were included. The uniquely mapping locations were used to generate the genome-wide intensity profiles, which were visualized using the IGV genome browser^48^. Promoter region was defined as ±2000 bp from the transcription start site. Data are available online in the GEO database (GSE98134). For ChIP-qPCR experiments, cells were fixed for 6 min with 1% PFA, and chromatin was sheared using Bioruptor Plus (Diagenode) for 45 cycles. Chromatin was immunopercipitated using 5 μg of Lsd1 (Biogenes, 20752), Hdac1 (Abcam, ab7028-50), Rcor1 (Abcam, ab32631), H3K4me2 (Diagenode, CS-035-100), or Glis1 antibody (Abcam, ab135724). Primer sequences used for qPCR experiment following ChIP are listed in Supplementary Table 5.

### Data analysis

All data were represented as positive standard error of the mean (SEM). Significance was calculated as stated:

1. by two-tailed Student’s *t*-test (Figs. 1c, 2e, 3d, 4f–h, 6a, b and 7b–d and Supplementary Figs. 1g, 2e, f, 3d, 4c, 5c, e, 7a, 8c, d, and g);
2. by two-way analysis of variance (ANOVA) (Figs. 1d, 2a, c, 4b–d, 6d, f and 7e and Supplementary Figs. 1d, e, h, 2c, 4e, 5a, f, 7c and 8h).

Heatmap was generated by centering and normalizing samples expression values with Cluster 3.0 and importing them to MeV viewer version 4.8.1. CSA of untreated muscle fibers was calculated using Image J software on the images taken from immunofluorescence assay using antibody directed against Dmd. Total of 2500 fibers were measured from five animals in each group. CSA measurement of regenerating fibers was performed by Image J software on Gomori-stained sections. Total of 1000 fibers was measured from five animals in each group. Nuclei counting was performed manually on Gomori-stained sections and normalized per regenerating muscle area calculated by Image J software. ORO fluorescence intensity was quantified using Image J software on immunofluorescence sections. Integrated density of the background measurement was subtracted from the integrated density of the selected area^49^.

### Data availability

RNA-seq and ChIP-seq data used in this study have been deposited in Gene Expression Omnibus (GEO) database under the accession codes GSE98133 and GSE98134, respectively.

## Electronic supplementary material

Supplementary Information

## References

[1] Karalaki M, Fili S, Philippou A, Koutsilieris M (2009). Muscle regeneration: cellular and molecular events. In Vivo.

[2] Wang YX, Rudnicki MA (2012). Satellite cells, the engines of muscle repair. Nat. Rev. Mol. Cell Biol..

[3] Seale P (2000). Pax7 is required for the specification of myogenic satellite cells. Cell.

[4] Mahdy MA, Lei HY, Wakamatsu J, Hosaka YZ, Nishimura T (2015). Comparative study of muscle regeneration following cardiotoxin and glycerol injury. Ann. Anat..

[5] Charge SB, Rudnicki MA (2004). Cellular and molecular regulation of muscle regeneration. Physiol. Rev..

[6] Bajard L (2006). A novel genetic hierarchy functions during hypaxial myogenesis: Pax3 directly activates Myf5 in muscle progenitor cells in the limb. Genes Dev..

[7] McKinnell IW (2008). Pax7 activates myogenic genes by recruitment of a histone methyltransferase complex. Nat. Cell Biol..

[8] Knapp JR (2006). Loss of myogenin in postnatal life leads to normal skeletal muscle but reduced body size. Development.

[9] Olson EN, Perry M, Schulz RA (1995). Regulation of muscle differentiation by the MEF2 family of MADS box transcription factors. Dev. Biol..

[10] Liu N (2014). Requirement of MEF2A, C, and D for skeletal muscle regeneration. Proc. Natl Acad. Sci. USA.

[11] Abmayr SM, Pavlath GK (2012). Myoblast fusion: lessons from flies and mice. Development.

[12] Sincennes MC, Brun CE, Rudnicki MA (2016). Concise review: epigenetic regulation of myogenesis in health and disease. Stem Cells Transl. Med..

[13] Moresi V, Marroncelli N, Adamo S (2015). New insights into the epigenetic control of satellite cells. World J. Stem Cells.

[14] Yin H (2013). MicroRNA-133 controls brown adipose determination in skeletal muscle satellite cells by targeting Prdm16. Cell Metab..

[15] Seale P (2008). PRDM16 controls a brown fat/skeletal muscle switch. Nature.

[16] Trajkovski M, Ahmed K, Esau CC, Stoffel M (2012). MyomiR-133 regulates brown fat differentiation through Prdm16. Nat. Cell Biol..

[17] Shi Y (2004). Histone demethylation mediated by the nuclear amine oxidase homolog LSD1. Cell.

[18] Metzger E (2005). LSD1 demethylates repressive histone marks to promote androgen-receptor-dependent transcription. Nature.

[19] Klose RJ, Zhang Y (2007). Regulation of histone methylation by demethylimination and demethylation. Nat. Rev. Mol. Cell Biol..

[20] Choi J (2014). Modulation of lysine methylation in myocyte enhancer factor 2 during skeletal muscle cell differentiation. Nucleic Acids Res..

[21] Scionti I (2017). LSD1 controls timely MyoD expression via MyoD core enhancer transcription. Cell Rep..

[22] Choi J (2010). Histone demethylase LSD1 is required to induce skeletal muscle differentiation by regulating myogenic factors. Biochem. Biophys. Res. Commun..

[23] Musri MM (2010). Histone demethylase LSD1 regulates adipogenesis. J. Biol. Chem..

[24] Duteil D (2014). LSD1 promotes oxidative metabolism of white adipose tissue. Nat. Commun..

[25] Chen Y (2016). Histone demethylase LSD1 promotes adipocyte differentiation through repressing wnt signaling. Cell Chem. Biol..

[26] Lepper C, Fan CM (2010). Inducible lineage tracing of Pax7-descendant cells reveals embryonic origin of adult satellite cells. Genesis.

[27] Zhu D (2014). Lysine-specific demethylase 1 regulates differentiation onset and migration of trophoblast stem cells. Nat. Commun..

[28] Duteil D (2016). Lsd1 ablation triggers metabolic reprogramming of brown adipose tissue. Cell Rep..

[29] Stoller JZ (2008). Cre reporter mouse expressing a nuclear localized fusion of GFP and beta-galactosidase reveals new derivatives of Pax3-expressing precursors. Genesis.

[30] Castex J (2017). Inactivation of Lsd1 triggers senescence in trophoblast stem cells by induction of Sirt4. Cell Death Dis..

[31] Tallquist MD, Weismann KE, Hellstrom M, Soriano P (2000). Early myotome specification regulates PDGFA expression and axial skeleton development. Development.

[32] Pasut A, Jones AE, Rudnicki MA (2013). Isolation and culture of individual myofibers and their satellite cells from adult skeletal muscle.. J. Vis. Exp.

[33] Gunther S (2013). Myf5-positive satellite cells contribute to Pax7-dependent long-term maintenance of adult muscle stem cells. Cell Stem Cell.

[34] Metzger E (2016). Assembly of methylated KDM1A and CHD1 drives androgen receptor-dependent transcription and translocation. Nat. Struct. Mol. Biol..

[35] Maekawa M, Yamanaka S (2011). Glis1, a unique pro-reprogramming factor, may facilitate clinical applications of iPSC technology. Cell Cycle.

[36] Maekawa M (2011). Direct reprogramming of somatic cells is promoted by maternal transcription factor Glis1. Nature.

[37] Sambeat A (2016). LSD1 interacts with Zfp516 to promote UCP1 transcription and brown fat program. Cell Rep..

[38] Duteil D (2017). Lsd1 prevents age-programed loss of beige adipocytes. Proc. Natl Acad. Sci. USA.

[39] Koopman R, Schaart G, Hesselink MK (2001). Optimisation of oil red O staining permits combination with immunofluorescence and automated quantification of lipids. Histochem. Cell Biol..

[40] Bookout, A. L., Cummins, C. L., Mangelsdorf, D. J., Pesola, J. M. & Kramer, M. F. High-throughput real-time quantitative reverse transcription PCR. *Curr. Protoc. Mol. Biol*. 10.1002/0471142727.mb1508s73 (2006). 10.1002/0471142727.mb1508s73 18265376

[41] Heinz S (2010). Simple combinations of lineage-determining transcription factors prime cis-regulatory elements required for macrophage and B cell identities. Mol. Cell.

[42] Robinson MD, McCarthy DJ, Smyth G (2010). K. edgeR: a bioconductor package for differential expression analysis of digital gene expression data. Bioinformatics.

[43] Zhang B, Kirov S, Snoddy J (2005). WebGestalt: an integrated system for exploring gene sets in various biological contexts. Nucleic Acids Res..

[44] Arrigoni L (2016). Standardizing chromatin research: a simple and universal method for ChIP-seq. Nucleic Acids Res..

[45] Langmead B, Trapnell C, Pop M, Salzberg SL (2009). Ultrafast and memory-efficient alignment of short DNA sequences to the human genome. Genome Biol..

[46] Zhang Y (2008). Model-based analysis of ChIP-Seq (MACS). Genome Biol..

[47] Shin H, Liu T, Manrai AK, Liu XS (2009). CEAS: cis-regulatory element annotation system. Bioinformatics.

[48] Thorvaldsdottir H, Robinson JT, Mesirov JP (2013). Integrative Genomics Viewer (IGV): high-performance genomics data visualization and exploration. Brief Bioinform..

[49] Burgess A (2010). Loss of human greatwall results in G2 arrest and multiple mitotic defects due to deregulation of the cyclin B-Cdc2/PP2A balance. Proc. Natl Acad. Sci. USA.

