# Supplementary material for: Lsd1 regulates skeletal muscle regeneration and directs the fate of satellite cells
Source: Nat Commun. 2018 Jan 25;9:366. doi: 10.1038/s41467-017-02740-5 (PMC5785540; doi:10.1038/s41467-017-02740-5)
Supplement: Supplementary file 1 — Supplementary Information [file 41467_2017_2740_MOESM1_ESM.pdf]

**Inventory of Supplementary Information**

**Lsd1 regulates skeletal muscle regeneration and directs the fate of satellite cells**

Milica Tosic, Anita Allen, Dominica Willmann, Christoph Lepper, Johnny Kim, Delphine Duteil, and Roland Schüle

**Supplementary Information contain:**

- **Supplementary Figures and Figure Legends 1-9**
- **Supplementary Tables 1-5**

## Supplementary Figures

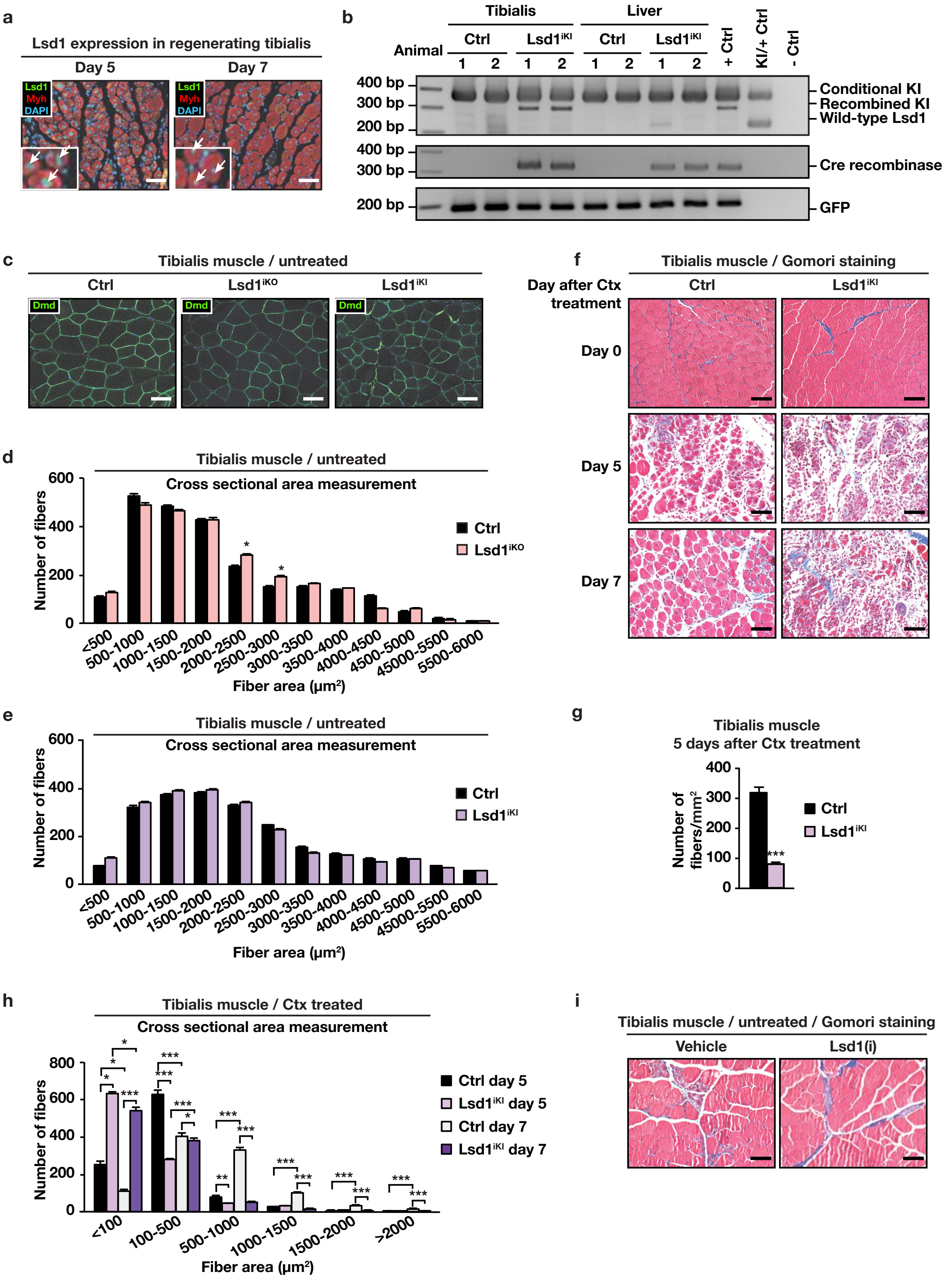


**Supplementary Figure 1. (a)** Immunofluorescence assay using antibodies directed against lysine specific demethylase (Lsd1, green) and pan-myosin heavy chain (Myh, red) on wild-type *tibialis* muscle sections 5 and 7 days after cardiotoxin (Ctx) treatment. Nuclei were stained with DAPI (blue).

Arrows indicate that regenerating centronuclei express Lsd1. **(b)** Genotyping of *tibialis* muscle and liver biopsies from control mice (Ctrl) and mice expressing enzymatically inactive Lsd1 mutant ($Lsd1^{iKI}$) for the presence of Lsd1 conditional or recombined knock-in (KI) alleles (upper panel), $Pax7^{Cre/ERT2}$ recombinase (middle panel), or green fluorescent protein (GFP) allele (bottom panel) by semi-quantitative PCR. **(c-e)** Analyses of untreated *tibialis* muscle sections from control mice (Ctrl), mice with Lsd1 ablation selectively in Pax7-positive satellite cells ($Lsd1^{iKO}$), or $Lsd1^{iKI}$ mice. **(c)** Immunofluorescence assay using antibody directed against dystrophin (Dmd, green). **(d, e)** Cross sectional area (CSA) measurement of fibers. Significance was calculated by two-way analysis of variance (ANOVA) test. **(f)** Gomori staining of representative *tibialis* muscle sections from Ctrl and $Lsd1^{iKI}$ mice 0, 5, and 7 days after Ctx treatment. **(g, h)** Analyses of regenerating centronuclear fibers from Ctrl and $Lsd1^{iKI}$ mice 5 or 7 days after Ctx treatment. **(g)** Number of fibers per area ($mm^2$). Significance was calculated by two-tailed Student's t-test. **(h)** CSA measurement of fibers. Significance was calculated by two-way ANOVA test. **(i)** Gomori staining of wild-type *tibialis* muscle treated with vehicle or Lsd1 inhibitor [Lsd1(i)] without Ctx injection. [(d, e), and (g, h): n=5; mean + SEM, * $p<0.05$, ** $p<0.01$, *** $p<0.001$; scale bars: (a, c) 50 µm, (f, i) 100 µm]

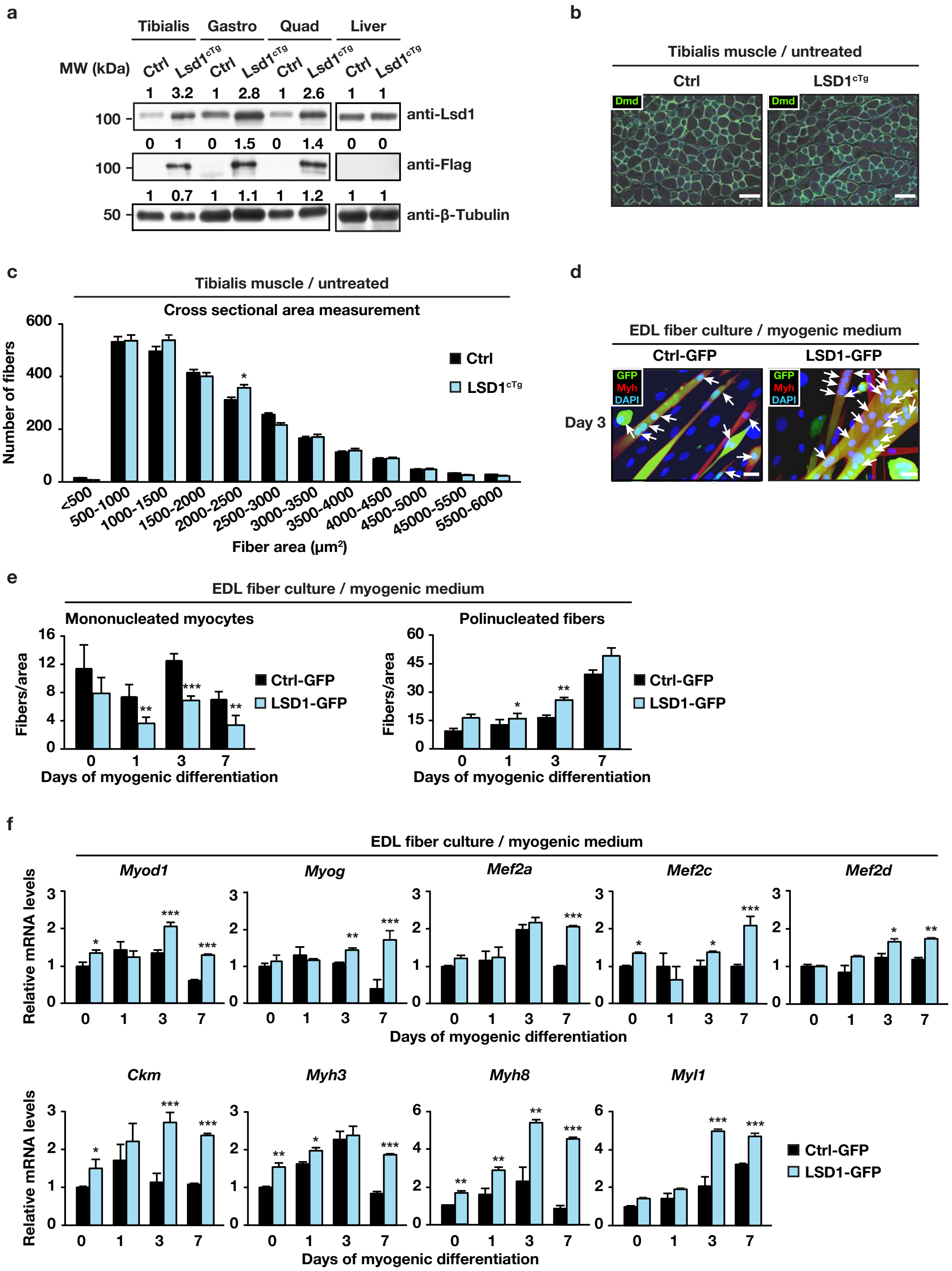


**Supplementary Figure 2. (a-c)** Analyses of untreated *tibialis* muscle from control (Ctrl) mice and mice overexpressing LSD1 selectively in Myf5-positive muscle precursors (LSD1$^{cTg}$). **(a)** Western blot analyses of endogenous Lsd1 and transgenic human LSD1 protein in *tibialis, gastrocnemius* (Gastro), or *quadriceps* (Quad) muscle and liver. Anti-Flag antibody was used to detect expression of ectopic human LSD1 protein. β-Tubulin served as a loading control. Numbers indicate relative protein levels. **(b)** Immunofluorescence assay using antibody directed against Dmd (green). **(c)** CSA

measurement of fibers. Significance was calculated by two-way ANOVA test. **(d)** Immunofluorescence assay using antibodies directed against green fluorescent protein (GFP, green) and Myh (red) on control (Ctrl-GFP) and LSD1 overexpressing satellite cells (LSD1-GFP) differentiated for 3 days in myogenic medium. Nuclei were stained with DAPI (blue). Arrows indicate higher number of nuclei in myofibers derived from LSD1-GFP compared to Ctrl-GFP satellite cells. **(e, f)** Analyses of Ctrl-GFP and LSD1-GFP expressing primary myofibers differentiated for 0, 1, 3, and 7 days in myogenic medium. **(e)** Quantification of mononucleated myocytes and polynucleated fibers, and **(f)** qRT-PCR analyses showing mRNA levels of indicated genes. Significance was calculated by two-tailed Student's t-test. [(c): n=5, (e, f): n=3; mean + SEM, * $p<0.05$, ** $p<0.01$, *** $p<0.001$; scale bars: (b) 50 µm, (d) 25 µm]

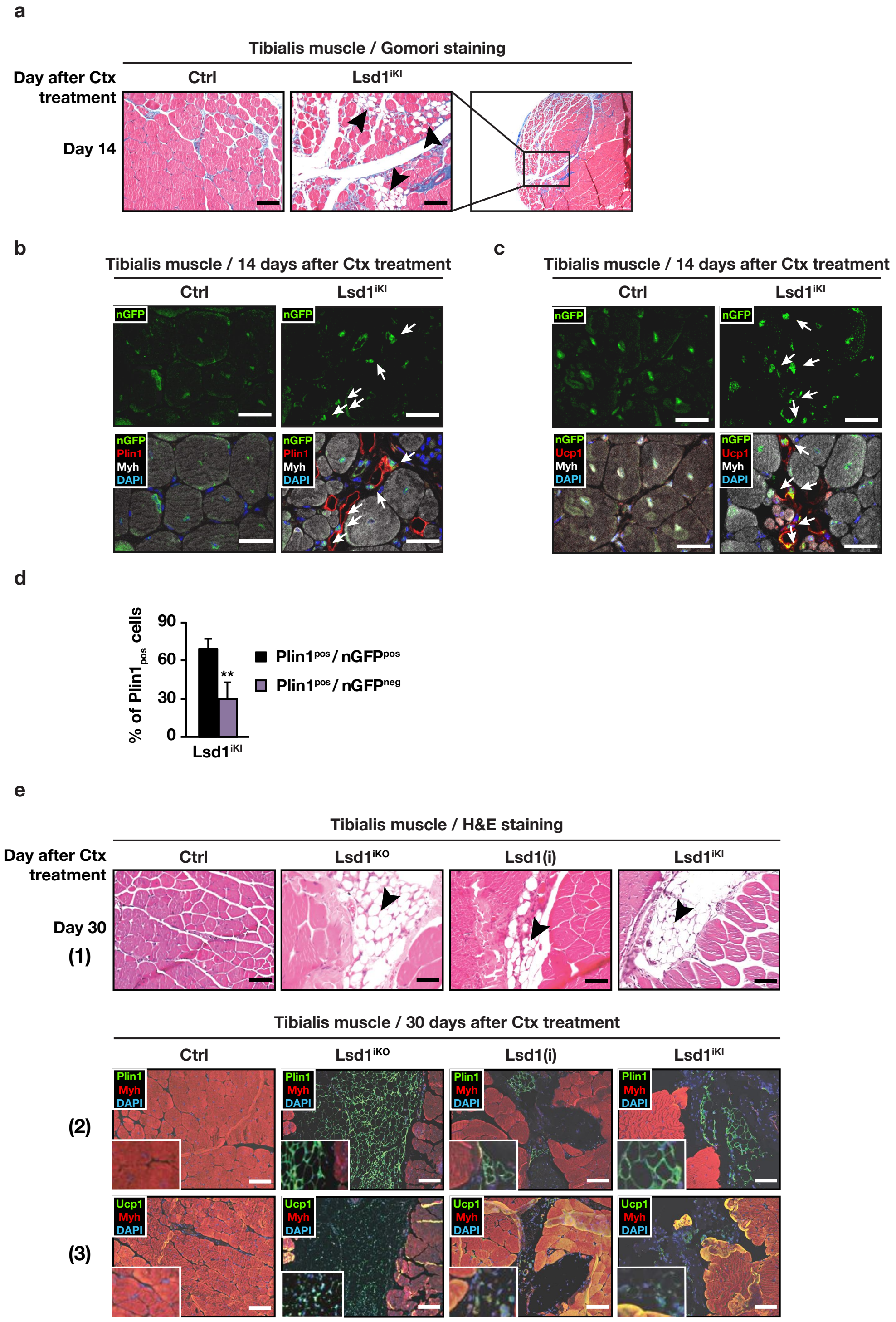


**Supplementary Figure 3. (a-d)** Analyses of Ctrl and Lsd1$^{iKI}$ *tibialis* muscle 14 days after Ctx treatment. **(a)** Gomori staining. Arrowheads indicate fat accumulation. **(b, c)** Immunofluorescence assay using antibodies directed against GFP (green), Myh (white) and **(b)** Plin1 (red), or **(c)** Ucp1 (red) as indicated. Nuclei were stained with DAPI (blue). Arrows indicate that adipocytes express nuclear GFP (nGFP) and therefore originate from satellite cells. **(d)** Percentage of Plin1-positive cells that express or do not express nGFP. Significance was calculated by two-tailed Student's t-test. **(e)** Hematoxylin and eosin (H&E) staining (1), and immunofluorescence assay using antibodies directed

against Plin1 (green) and Myh (red) (2) or Ucp1 (green) and Myh (red) (3) on representative sections from Ctrl, $Lsd1^{iKO}$, Lsd1(i), and $Lsd1^{iKI}$ *tibialis* muscle 30 days after Ctx treatment. Nuclei were stained with DAPI (blue). Arrowheads indicate fat accumulation. [(d) n=6; mean + SEM ** p<0.01; scale bars: (a, e) 100 µm, (b, c) 50 µm].

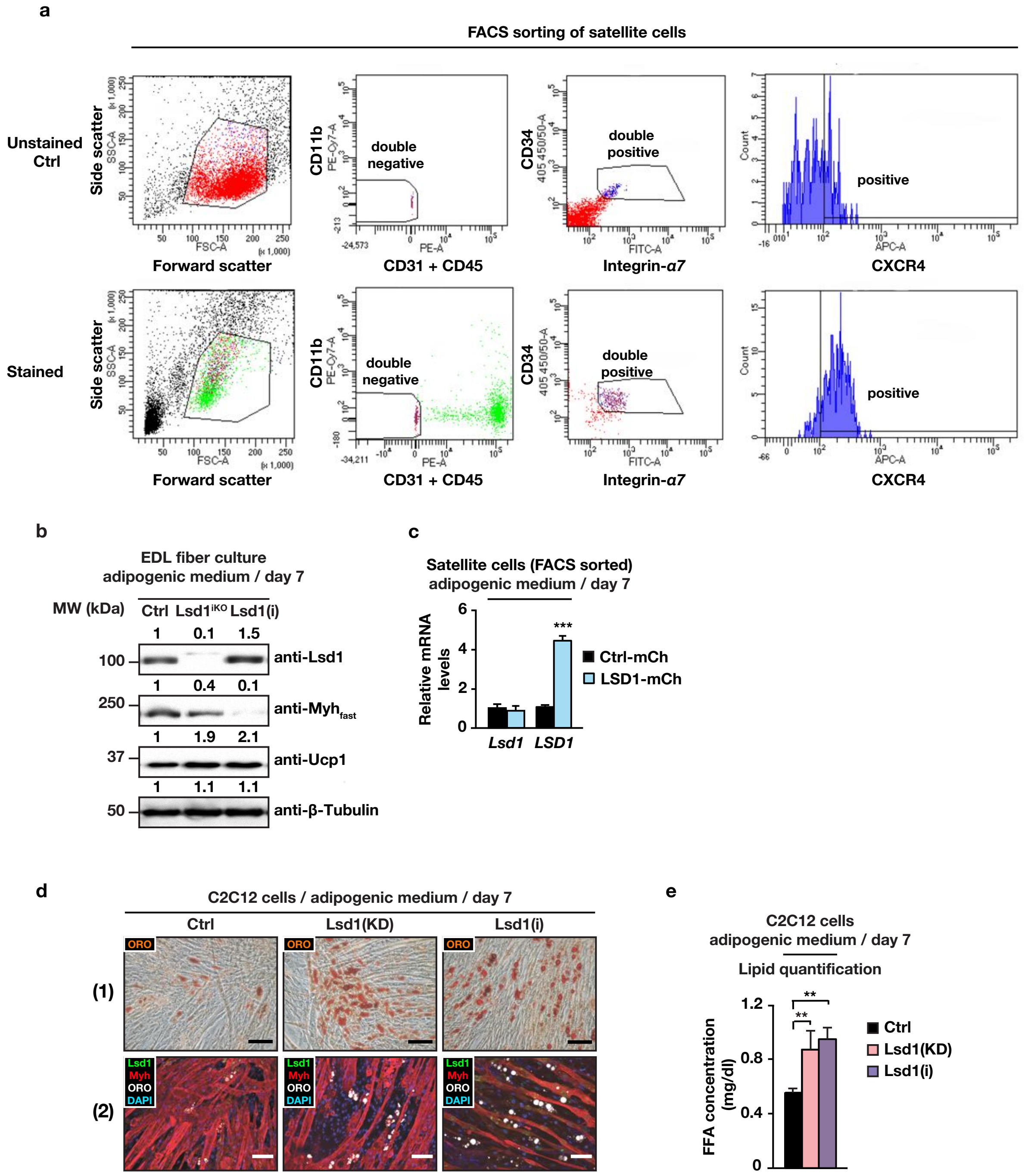


**Supplementary Figure 4. (a)** Gating strategy used for FACS sorting of satellite cells. CD11b, CD45, and CD31 were used to exclude leukocytes and endothelial cells, whereas CD34, Integrin-α7, and CXCR4 were used as positive markers for satellite cells. **(b)** Western blot analyses of Lsd1, Myh, and Ucp1 protein levels in control (Ctrl), Lsd1-ablated ($Lsd1^{iKO}$), or Lsd1 inhibitor-treated [Lsd1(i)] satellite cells differentiated for 7 days in adipogenic medium. β-Tubulin served as a loading control. Numbers indicate relative protein levels. **(c)** qRT-PCR analyses showing relative transcript levels of endogenous *Lsd1* and ectopically expressed human *LSD1*. Significance was calculated by two-tailed Student's t-test. **(d-g)** Analyses of Ctrl, Lsd1 knock-down [Lsd1(KD)], and Lsd1(i)-treated C2C12 cells. **(d)** Oil red O (ORO) staining (1) and immunofluorescence assay using antibodies directed

against Lsd1 (green) and Myh (red) (2) at day 7 of adipogenic differentiation. ORO fluorescence is depicted in white. Nuclei were stained with DAPI (blue). **(e)** Quantification of lipid content by measuring free fatty acid (FFA) concentration (mg/dl) at day 7 of adipogenic differentiation. Significance was calculated by two-way ANOVA test. [(c): n=4, (e): n=6; mean + SEM * $p<0.05$, ** $p<0.01$, *** $p<0.001$; scale bars: (d): (1) 50 µm; (2) 100 µm].

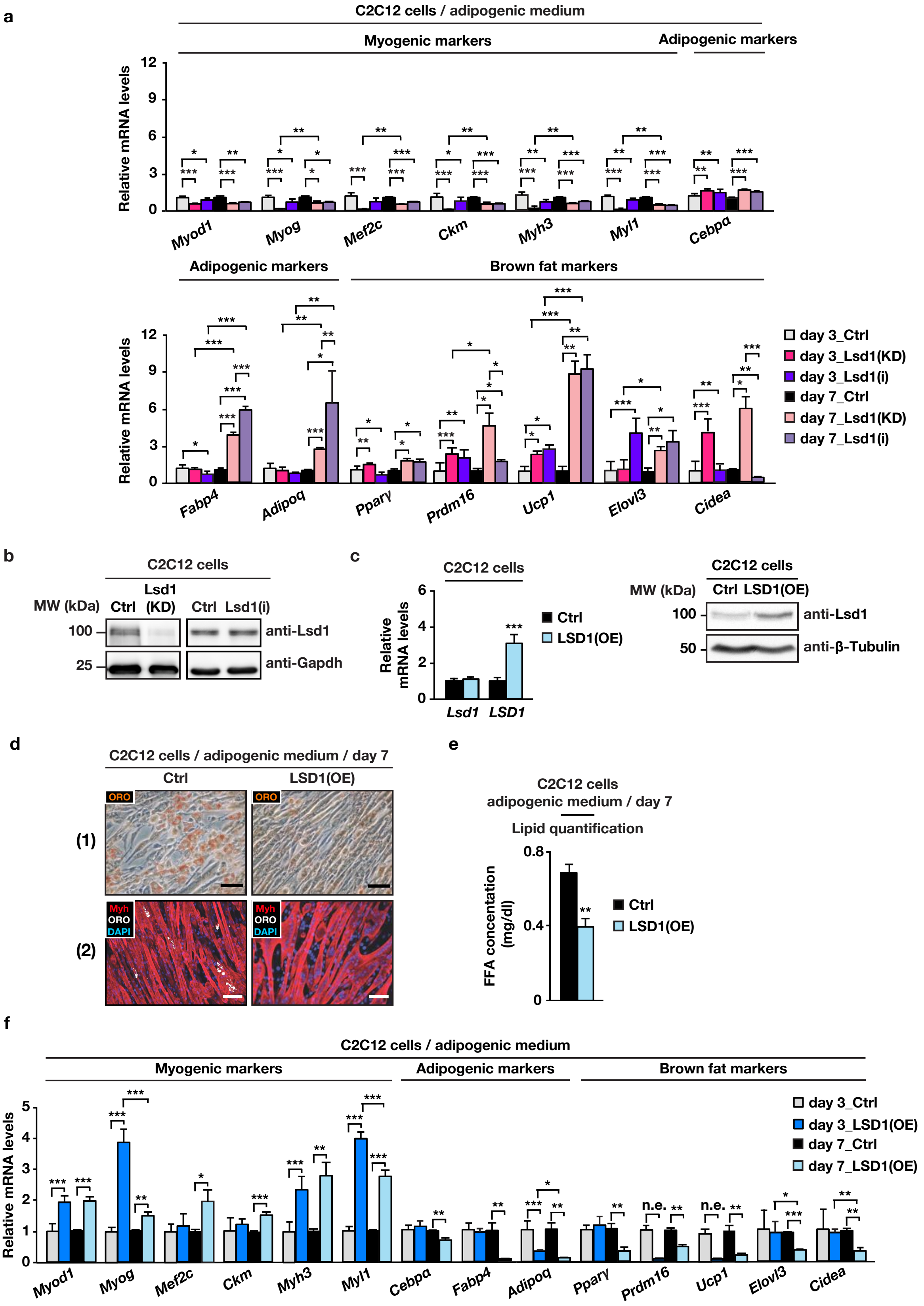


**Supplementary Figure 5. (a)** qRT-PCR analysis showing relative transcript levels of indicated genes at day 3 and 7 of adipogenic differentiation. Significance was calculated by two-way ANOVA test. **(b)** Western blot analysis of Lsd1 protein levels. Gapdh served as a loading control. **(c-f)** Analyses of

Ctrl and LSD1(OE) C2C12 cells. **(c)** qRT-PCR analysis showing relative transcript levels of endogenous *Lsd1* and ectopically expressed human *LSD1* (left panel). Significance was calculated by two-tailed Student's t-test. Western blot analysis of control (Ctrl) and LSD1 overexpressing [LSD1(OE)] C2C12 cells (right panel). β-Tubulin served as a loading control. **(d)** ORO staining (1) and immunofluorescence assay using antibody directed against Myh (red) (2) at day 7 of adipogenic differentiation. ORO fluorescence is depicted in white. Nuclei were stained with DAPI (blue). **(e)** Quantification of lipid content by measuring free fatty acid (FFA) concentration (mg/dl) at day 7 of adipogenic differentiation**.** Significance was calculated by two-tailed Student's t-test. **(f)** qRT-PCR analysis showing relative transcript levels of indicated genes at day 3 and 7 of adipogenic differentiation. Significance was calculated by two-way ANOVA test. [(a, c, e, f): n=6; mean + SEM * p<0.05, ** p<0.01, *** p<0.001; scale bars: (d): (1) 50 μm; (2) 100 μm].

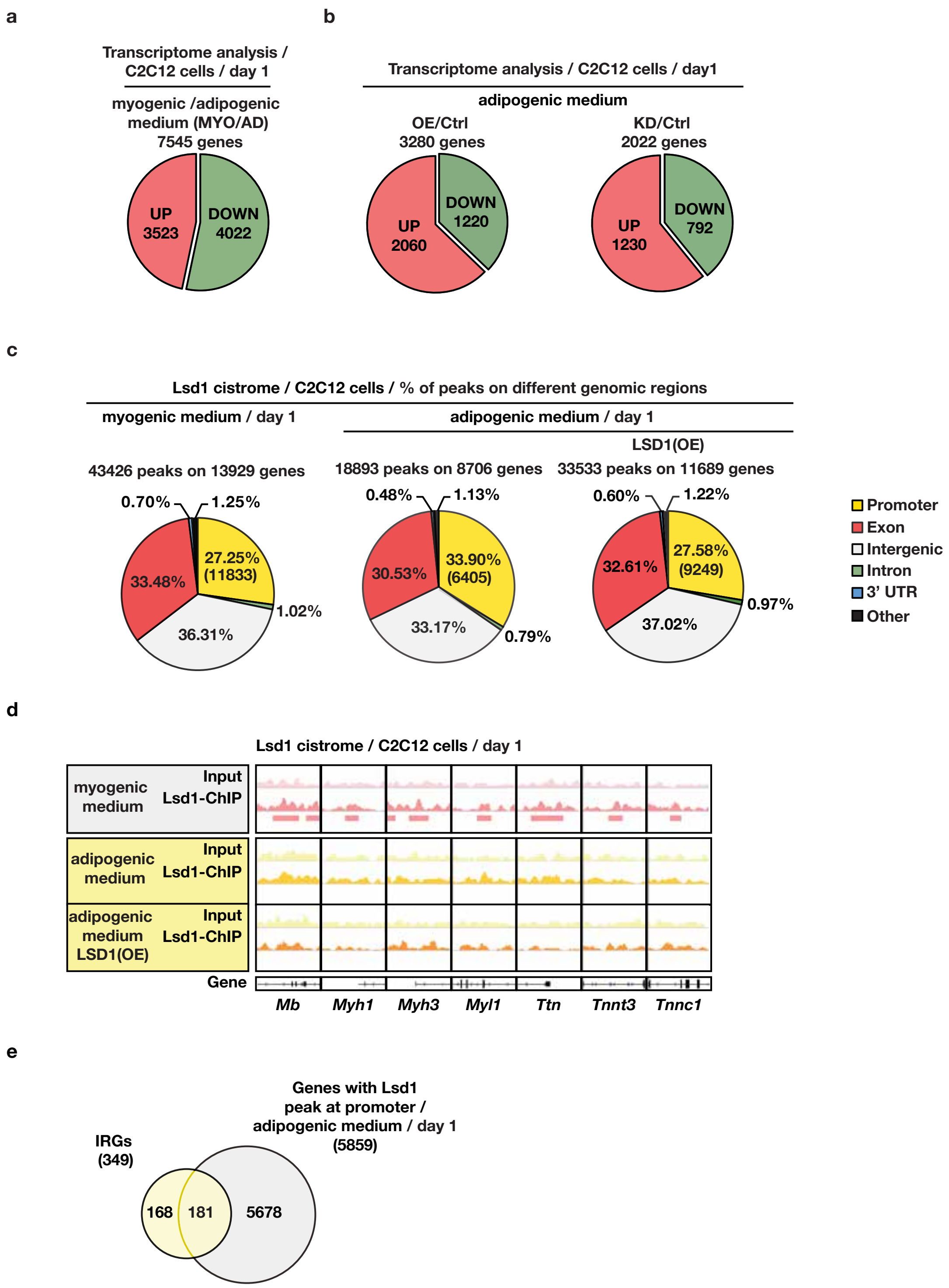


**Supplementary figure 6. (a, b)** Pie charts depicting total number of differentially expressed genes (DEGs) and number of upregulated and downregulated genes obtained by RNA-seq analyses of **(a)** wild type C2C12 cells differentiated for 1 day in myogenic versus adipogenic medium (MYO/AD) and **(b)** Lsd1 overexpressing versus control C2C12 cells (OE/Ctrl) or Lsd1 knock-down and control C2C12 cells differentiated for 1 day in adipogenic medium (KD/Ctrl). **(c)** Pie charts depicting total number of high-confidence Lsd1 peaks, number of corresponding genes, and percentage of Lsd1 peaks at indicated genomic regions obtained by ChIP-seq analyses of C2C12 cells differentiated for 1

day in myogenic or adipogenic medium, or in LSD1(OE) C2C12 cells differentiated for 1 day in adipogenic medium. **(d)** ChIP-seq tracks depicting Lsd1 chromatin occupancy at indicated promoters in wild-type C2C12 cells differentiated for 1 day in myogenic and adipogenic medium or LSD1 overexpressing [LSD1(OE)] C2C12 cells differentiated for 1 day in adipogenic medium. Identified peaks are marked with bars. **(e)** Overlap of all 349 IRGs (yellow) obtained from RNA-seq analysis and genes with Lsd1 promoter occupancy (dark gray) obtained from ChIP-seq analyses of C2C12 cells differentiated for 1 day in adipogenic medium.

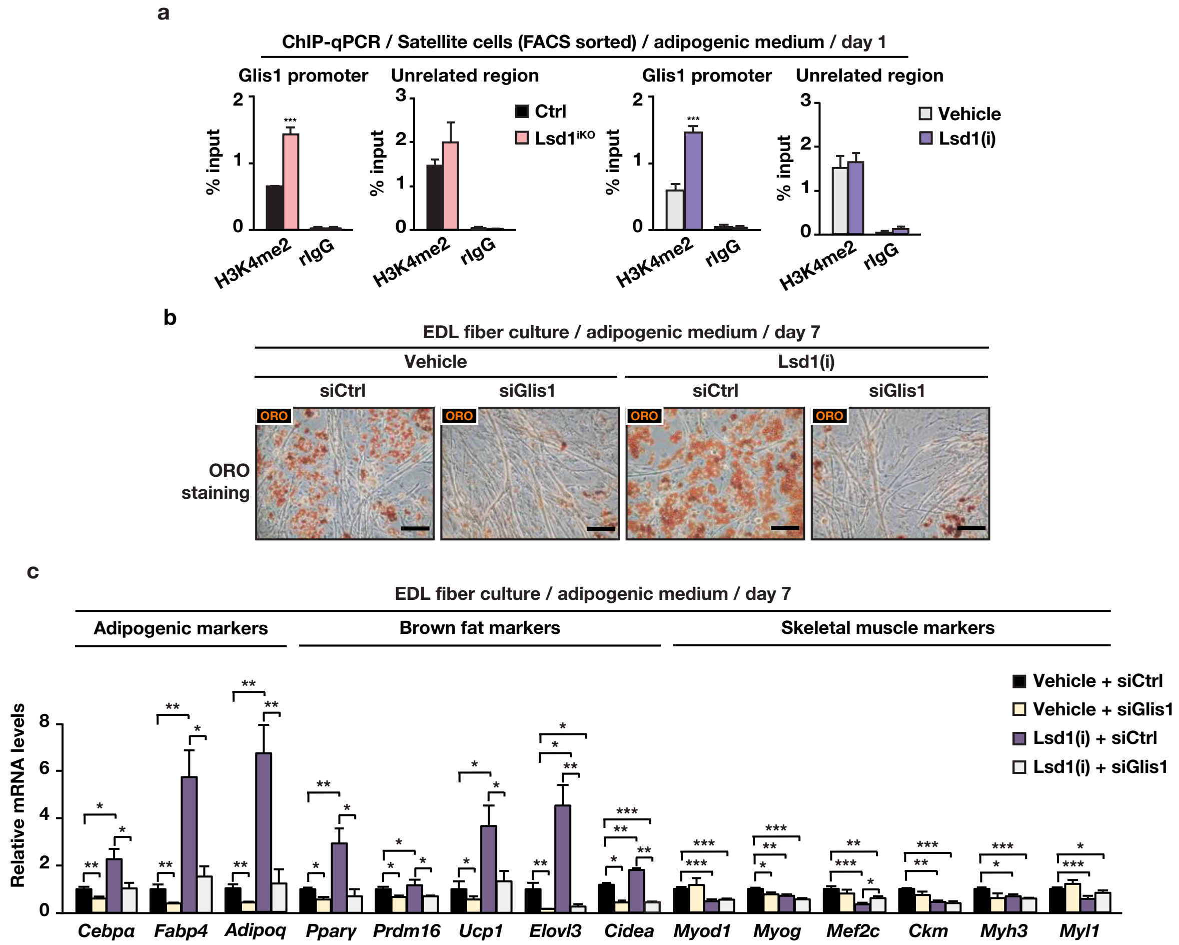


**Supplementary Figure 7. (a)** ChIP-qPCR analyses using antibodies directed against dimethylated histone 3 lysine 4 (H3K4me2), or rabbit immunoglobulin G (rIgG) in FACS sorted control (Ctrl) and Lsd1$^{iKO}$ satellite cells (left panel), or vehicle and Lsd1(i)-treated satellite cells (right panel) differentiated for 1 day in adipogenic medium. Immunoprecipitated chromatin was quantified by qPCR using primers flanking Lsd1-binding sites at the promoter of *Glis1* gene or unrelated region. Significance was calculated by two-tailed Student's t-test. **(b, c)** Analyses of Ctrl and Lsd1(i)-treated EDL fiber culture cells transfected with control siRNA (siCtrl) or siRNA directed against *Glis1* (siGlis1) and differentiated for 7 days in adipogenic medium. **(b)** ORO staining. **(c)** qRT-PCR analysis showing relative transcript levels of indicated genes. Significance was calculated by two-way ANOVA test. [(a, c): n=3; mean + SEM * p<0.05, ** p<0.01, *** p<0.001; scale bars: (b) 50 µm]

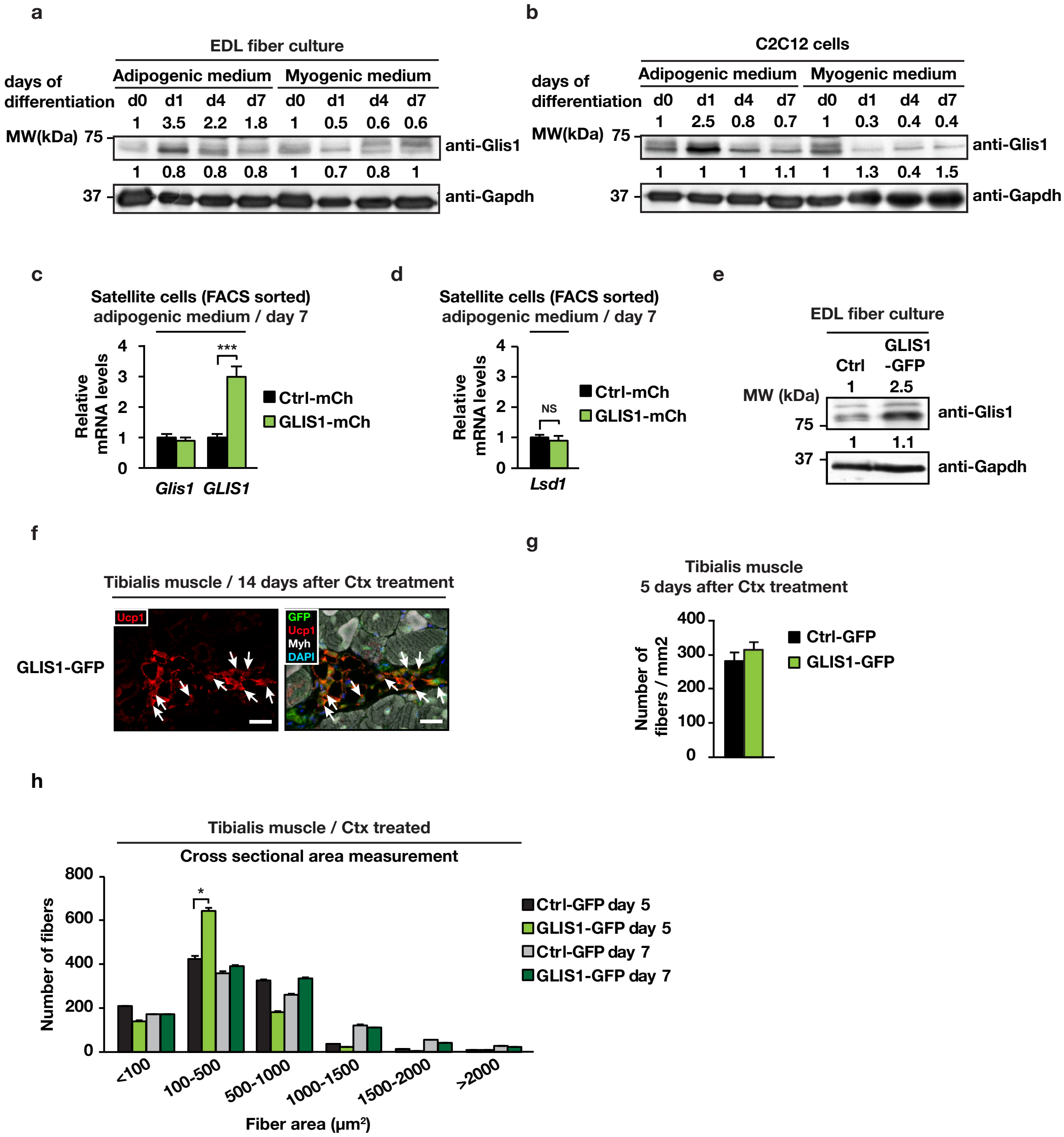


**Supplementary Figure 8. (a, b)** Western blot analysis of Glis1 protein levels in **(a)** satellite and **(b)** C2C12 cells differentiated for 0, 1, 3, 5, or 7 days under adipogenic and myogenic conditions. Gapdh served as a loading control. Numbers indicate relative protein levels. **(c, d)** Analyses of control (Ctrl-mCh) and GLIS1 overexpressing (GLIS1-mCh) satellite cells. **(c)** qRT-PCR analysis showing relative transcript levels of endogenous *Glis1* and ectopic human *GLIS1* transcript. Significance was calculated by two-tailed Student's t-test. **(d)** qRT-PCR analysis showing relative transcript levels of *Lsd1*. Significance was calculated by two-tailed Student's t-test. **(e)** Western blot analysis showing Glis1 protein levels in Ctrl-GFP and GLIS1-GFP satellite cells. Gapdh served as a loading control. Numbers indicate relative protein levels. **(f)** Immunofluorescence assay using antibodies directed against GFP (green), Ucp1 (red), and Myh (white) as indicated. Nuclei were stained with DAPI (blue). Arrows indicate that Ucp1-positive adipocytes express GFP. **(g, h)** Analyses of regenerating

centronuclear fibers in *tibialis* muscle of wild-type mice injected with Ctrl-GFP or GLIS1-GFP adenovirus 5 or 7 days after Ctx treatment. **(g)** Number of fibers per area ($mm^2$). Significance was calculated by two-tailed Student's t-test. **(h)** CSA measurement of fibers. Significance was calculated by two-way ANOVA test. [(c, d): n=3, (f-h): n=5; mean + SEM NS = non-significant, * p<0.05, ** p<0.01, *** p<0.001; scale bars: (f) 50 µm]

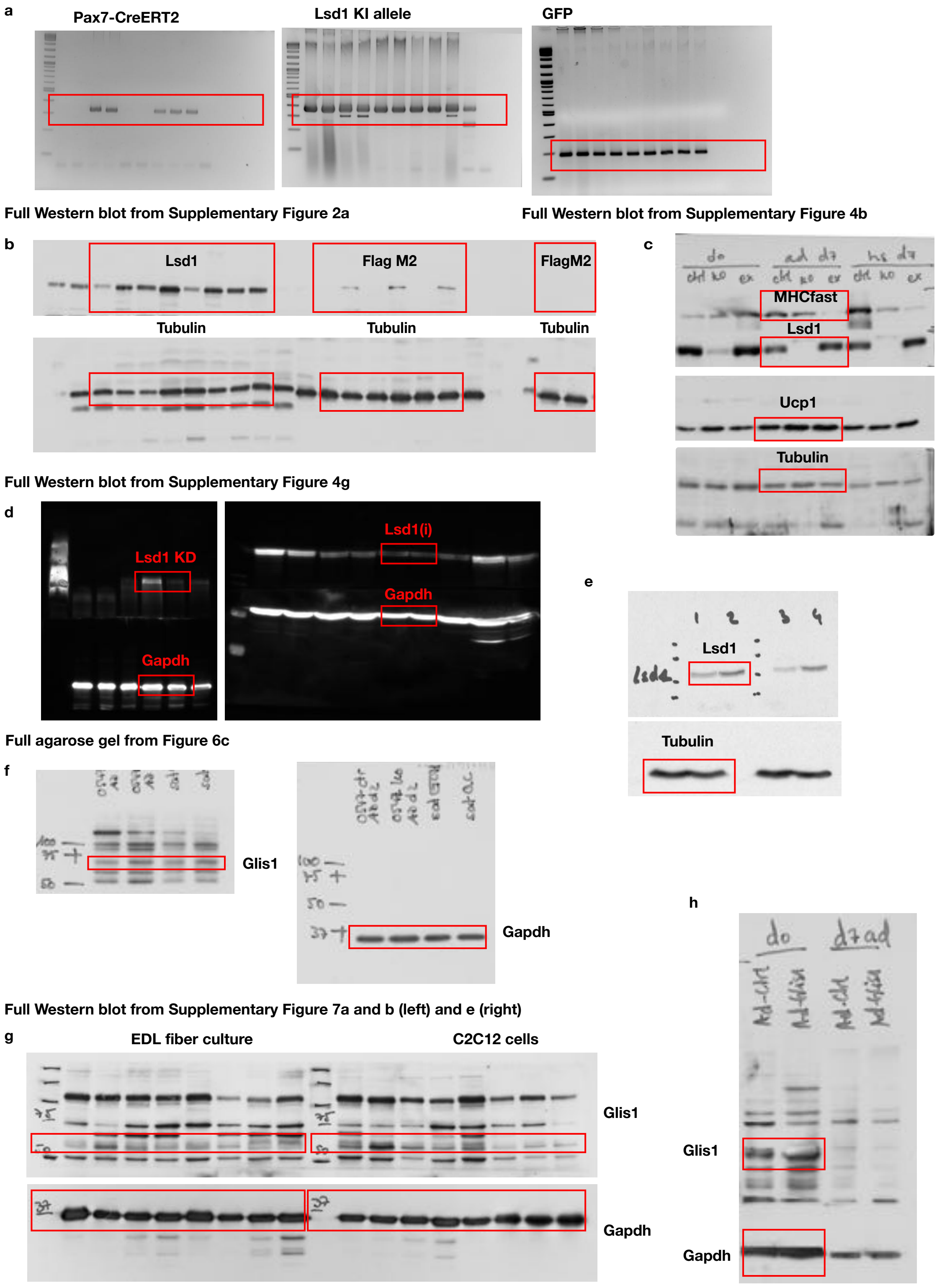


**Supplementary Figure 9.** Full gels and Western blots

**Supplementary Table 1:** List of 275 inversely regulated genes obtained from RNA-seq analyses in C2C12 cells at day 1 of adipogenic differentiation that are upregulated upon LSD1 overexpression and downregulated upon Lsd1 knock-down.

| | Fold Change of Expression | | |
|---|---|---|---|
| ***Gene name*** | **Myo/Ad** | **Ad:siCTRL vs. siLsd1** | **Ad:Ctrl vs. LSD1(OE)** |
| *1190002N15Rik* | 1.65 | 0.59 | 1.41 |
| *2010111I01Rik* | 4.05 | 0.71 | 1.84 |
| *A930016O22Rik* | 14.87 | 0.31 | 7.56 |
| *Abca5* | 2.91 | 0.77 | 1.94 |
| *Abcd2* | 2.10 | 0.65 | 2.59 |
| *Abl2* | 2.72 | 0.74 | 1.53 |
| *Ablim3* | 19.61 | 0.46 | 10.55 |
| *Acsl3* | 2.20 | 0.66 | 1.53 |
| *Acta2* | 7.92 | 0.54 | 2.88 |
| *Actc1* | 68.61 | 0.18 | 37.68 |
| *Actn3* | 2.81 | 0.56 | 2.29 |
| *Adam19* | 4.29 | 0.77 | 2.13 |
| *Adamts5* | 18.39 | 0.53 | 3.56 |
| *Ago2* | 4.13 | 0.69 | 3.51 |
| *Ajuba* | 4.31 | 0.67 | 1.75 |
| *Akap6* | 7.07 | 0.62 | 2.85 |
| *Akt2* | 1.54 | 0.76 | 1.50 |
| *Alg10b* | 1.83 | 0.61 | 1.75 |
| *Alpk2* | 2.95 | 0.49 | 1.76 |
| *Alpk3* | 23.28 | 0.50 | 11.01 |
| *Ammecr1* | 2.70 | 0.56 | 2.09 |
| *Ank1* | 4.05 | 0.62 | 3.06 |
| *Ankrd10* | 1.60 | 0.70 | 1.54 |
| *Ankrd44* | 2.22 | 0.67 | 1.49 |
| *Ankrd50* | 1.96 | 0.77 | 1.86 |
| *Ankrd52* | 2.33 | 0.72 | 1.73 |
| *Arhgap28* | 2.31 | 0.72 | 1.71 |
| *Arid5b* | 5.52 | 0.70 | 3.95 |
| *Arpp21* | 40.32 | 0.14 | 9.75 |
| *Arx* | 29.92 | 0.14 | 13.91 |
| *Atp13a3* | 2.20 | 0.66 | 1.71 |
| *Atp1b1* | 2.06 | 0.68 | 1.70 |
| *Atp2a1* | 6.31 | 0.45 | 5.87 |
| *Baz2a* | 1.48 | 0.76 | 1.47 |
| *Bbx* | 1.42 | 0.75 | 1.55 |
| *Bcor* | 2.13 | 0.77 | 1.37 |
| *Bin1* | 2.35 | 0.69 | 1.81 |
| *Birc6* | 1.40 | 0.70 | 1.32 |
| *Bmpr2* | 6.44 | 0.60 | 4.15 |
| *Brd4* | 1.80 | 0.71 | 1.65 |
| *Brwd1* | 3.03 | 0.71 | 2.23 |

| | Fold Change of Expression | | |
|---|---|---|---|
| **Gene name** | **Myo/Ad** | **Ad:siCTRL vs. siLsd1** | **Ad:Ctrl vs. LSD1(OE)** |
| *C78339* | 3.03 | 0.68 | 2.17 |
| *Cbl* | 5.02 | 0.62 | 4.55 |
| *Ccdc141* | 9.00 | 0.45 | 4.11 |
| *Ccdc186* | 3.37 | 0.71 | 2.49 |
| *Ccdc23* | 1.80 | 0.64 | 1.79 |
| *Ccdc88c* | 10.23 | 0.53 | 6.98 |
| *Ccnt1* | 2.03 | 0.71 | 1.81 |
| *Cd36* | 67.91 | 0.33 | 31.27 |
| *Cdk19* | 1.39 | 0.69 | 1.76 |
| *Cdk5r1* | 2.81 | 0.48 | 2.15 |
| *Celf2* | 2.68 | 0.69 | 1.68 |
| *Chst15* | 8.32 | 0.61 | 2.99 |
| *Ckb* | 2.85 | 0.74 | 1.49 |
| *Ckm* | 18.02 | 0.31 | 9.43 |
| *Cnn3* | 1.70 | 0.75 | 1.53 |
| *Cnot6* | 1.63 | 0.72 | 1.57 |
| *Cnot6l* | 3.23 | 0.67 | 2.61 |
| *Cobl* | 6.06 | 0.55 | 3.72 |
| *Cog5* | 1.83 | 0.77 | 1.72 |
| *Col12a1* | 2.25 | 0.75 | 2.07 |
| *Copg2* | 1.58 | 0.75 | 1.75 |
| *Crkl* | 1.35 | 0.77 | 1.46 |
| *Cux1* | 2.31 | 0.66 | 2.01 |
| *Cxadr* | 5.82 | 0.43 | 3.33 |
| *D830031N03Rik* | 5.46 | 0.73 | 3.21 |
| *Ddc* | 5.04 | 0.39 | 5.57 |
| *Ddi2* | 1.34 | 0.75 | 1.41 |
| *Deptor* | 9.21 | 0.56 | 7.97 |
| *Dgkd* | 2.79 | 0.65 | 1.71 |
| *Dlgap4* | 2.33 | 0.70 | 1.59 |
| *Dll1* | 3.54 | 0.43 | 2.10 |
| *Dok7* | 16.66 | 0.34 | 5.93 |
| *Dpysl3* | 9.29 | 0.66 | 5.31 |
| *Dusp27* | 8.04 | 0.52 | 3.19 |
| *Dusp4* | 3.18 | 0.75 | 1.54 |
| *Dvl3* | 2.17 | 0.69 | 1.70 |
| *Dyrk2* | 4.83 | 0.75 | 2.93 |
| *E130310I04Rik* | 7.41 | 0.39 | 7.14 |
| *Enah* | 3.51 | 0.70 | 2.25 |
| *Epha7* | 2.89 | 0.65 | 4.44 |
| *Ephb2* | 1.61 | 0.71 | 2.56 |
| *Erbb2ip* | 1.99 | 0.69 | 1.46 |
| *Erbb3* | 27.45 | 0.36 | 10.49 |
| *Ets1* | 1.37 | 0.69 | 1.71 |
| *Eya4* | 2.54 | 0.48 | 3.10 |

| | Fold Change of Expression | | |
|---|---|---|---|
| ***Gene name*** | **Myo/Ad** | **Ad:siCTRL vs. siLsd1** | **Ad:Ctrl vs. LSD1(OE)** |
| *Fam13c* | 4.53 | 0.73 | 2.62 |
| *Fam160a1* | 1.60 | 0.64 | 1.58 |
| *Fam214a* | 2.52 | 0.68 | 1.83 |
| *Farp1* | 2.21 | 0.71 | 1.41 |
| *Fat1* | 1.37 | 0.66 | 1.66 |
| *Fbxo17* | 4.61 | 0.56 | 2.29 |
| *Fbxo32* | 14.76 | 0.43 | 4.80 |
| *Fgf21* | 10.41 | 0.44 | 71.95 |
| *Fhod3* | 2.26 | 0.63 | 1.65 |
| *Fndc5* | 27.16 | 0.40 | 8.13 |
| *Foxk1* | 1.95 | 0.72 | 1.98 |
| *Foxo4* | 1.73 | 0.70 | 1.51 |
| *Frmd4b* | 20.59 | 0.29 | 18.82 |
| *Frmpd1* | 3.08 | 0.61 | 2.34 |
| *Gan* | 4.93 | 0.60 | 4.62 |
| *Glt28d2* | 1.74 | 0.57 | 1.83 |
| *Gpam* | 2.15 | 0.65 | 2.35 |
| *Gpt2* | 1.59 | 0.70 | 2.08 |
| *H19* | 3.13 | 0.42 | 3.34 |
| *Hdac5* | 4.62 | 0.77 | 1.64 |
| *Hdgfrp3* | 3.64 | 0.45 | 8.30 |
| *Hectd1* | 2.11 | 0.71 | 1.70 |
| *Herc1* | 2.06 | 0.76 | 1.56 |
| *Hfe2* | 12.17 | 0.47 | 5.69 |
| *Hip1* | 4.25 | 0.68 | 2.52 |
| *Hipk2* | 2.79 | 0.67 | 3.05 |
| *Hoxc11* | 1.70 | 0.70 | 1.73 |
| *Hrc* | 21.79 | 0.48 | 9.57 |
| *Iffo1* | 2.01 | 0.68 | 1.50 |
| *Igf2* | 7.70 | 0.57 | 4.69 |
| *Igfn1* | 10.68 | 0.24 | 20.34 |
| *Inppl1* | 2.67 | 0.77 | 1.67 |
| *Jarid2* | 1.48 | 0.71 | 1.45 |
| *Kalrn* | 8.15 | 0.74 | 5.42 |
| *Kcnk13* | 3.74 | 0.24 | 2.75 |
| *Kdm5b* | 3.76 | 0.63 | 2.89 |
| *Klhl13* | 8.97 | 0.54 | 8.60 |
| *Klhl41* | 40.18 | 0.50 | 15.04 |
| *Lama1* | 10.38 | 0.57 | 260.04 |
| *Ldb3* | 10.17 | 0.45 | 6.49 |
| *Lmod3* | 16.09 | 0.45 | 8.66 |
| *Lnpep* | 5.36 | 0.58 | 4.11 |
| *Lpar4* | 2.30 | 0.53 | 5.29 |
| *Lrch2* | 1.99 | 0.71 | 1.82 |
| *Lyst* | 2.52 | 0.71 | 1.93 |

| | Fold Change of Expression | | |
|---|---|---|---|
| ***Gene name*** | **Myo/Ad** | **Ad:siCTRL vs. siLsd1** | **Ad:Ctrl vs. LSD1(OE)** |
| *Maml3* | 2.48 | 0.67 | 1.66 |
| *Map3k9* | 2.25 | 0.38 | 5.92 |
| *Mb* | 8.83 | 0.56 | 11.47 |
| *Mef2a* | 3.30 | 0.64 | 2.99 |
| *Mef2c* | 9.28 | 0.51 | 5.00 |
| *Mef2d* | 2.16 | 0.71 | 1.74 |
| *Megf10* | 5.15 | 0.62 | 3.62 |
| *Mib1* | 3.76 | 0.66 | 3.35 |
| *Mical2* | 3.44 | 0.70 | 1.32 |
| *Mical3* | 4.30 | 0.68 | 3.71 |
| *Mid1* | 3.69 | 0.73 | 2.22 |
| *Mir5114* | 2.32 | 0.65 | 2.69 |
| *Mir675* | 3.15 | 0.38 | 3.38 |
| *Mlxip* | 3.10 | 0.67 | 3.26 |
| *Mob1b* | 2.02 | 0.63 | 1.68 |
| *Msi2* | 2.56 | 0.70 | 2.18 |
| *Mtss1l* | 2.76 | 0.68 | 3.01 |
| *Musk* | 1.41 | 0.64 | 2.34 |
| *Mybpc1* | 46.49 | 0.15 | 61.03 |
| *Mycl* | 1.34 | 0.48 | 1.77 |
| *Myh1* | 24.01 | 0.52 | 15.33 |
| *Myh2* | 19.15 | 0.44 | 10.92 |
| *Myh3* | 13.49 | 0.45 | 12.86 |
| *Myh4* | 14.46 | 0.30 | 22.24 |
| *Myh7* | 26.60 | 0.21 | 22.21 |
| *Myh7b* | 5.89 | 0.43 | 4.84 |
| *Myh8* | 30.18 | 0.21 | 19.29 |
| *Myl1* | 4.64 | 0.49 | 5.85 |
| *Mylk* | 3.87 | 0.41 | 4.43 |
| *Mylk4* | 42.50 | 0.15 | 22.70 |
| *Mylpf* | 6.44 | 0.55 | 3.90 |
| *Myog* | 6.18 | 0.37 | 5.95 |
| *Myom1* | 48.02 | 0.55 | 18.94 |
| *Myom3* | 28.28 | 0.45 | 15.86 |
| *Naca* | 3.95 | 0.76 | 2.78 |
| *Nckap5l* | 2.29 | 0.67 | 1.53 |
| *Ncoa1* | 1.92 | 0.75 | 2.06 |
| *Ndst4* | 1.54 | 0.58 | 3.50 |
| *Neb* | 17.03 | 0.50 | 12.83 |
| *Nek7* | 1.40 | 0.69 | 1.37 |
| *Nol4* | 2.82 | 0.32 | 1.60 |
| *Nr1d1* | 6.47 | 0.73 | 4.90 |
| *Nr1d2* | 12.58 | 0.58 | 5.25 |
| *Obscn* | 17.97 | 0.37 | 8.97 |
| *Palld* | 6.65 | 0.62 | 4.91 |

| | Fold Change of Expression | | |
|---|---|---|---|
| ***Gene name*** | **Myo/Ad** | **Ad:siCTRL vs. siLsd1** | **Ad:Ctrl vs. LSD1(OE)** |
| *Palm2* | 7.68 | 0.58 | 3.02 |
| *Pcdh18* | 2.14 | 0.67 | 2.17 |
| *Pcgf3* | 1.74 | 0.67 | 1.52 |
| *Pdcd4* | 2.61 | 0.64 | 1.97 |
| *Pdlim3* | 4.46 | 0.60 | 3.29 |
| *Peg10* | 2.61 | 0.42 | 1.54 |
| *Peg3* | 2.70 | 0.51 | 2.06 |
| *Peg3os* | 2.00 | 0.55 | 1.49 |
| *Pgm5* | 60.92 | 0.48 | 17.52 |
| *Pik3r3* | 1.50 | 0.63 | 1.46 |
| *Pim1* | 2.32 | 0.73 | 1.74 |
| *Pkia* | 4.62 | 0.61 | 2.52 |
| *Plcg1* | 1.85 | 0.73 | 1.54 |
| *Ppapdc3* | 11.35 | 0.26 | 4.82 |
| *Ppargc1b* | 2.96 | 0.54 | 2.16 |
| *Ppfia4* | 8.17 | 0.43 | 6.25 |
| *Prkaa2* | 13.95 | 0.48 | 10.49 |
| *Prkar2a* | 1.37 | 0.63 | 1.54 |
| *Prrc2b* | 2.10 | 0.75 | 1.34 |
| *Prune2* | 1.51 | 0.69 | 2.14 |
| *Pstpip2* | 1.89 | 0.73 | 1.53 |
| *Ptpn21* | 3.79 | 0.74 | 1.58 |
| *Qk* | 2.05 | 0.72 | 1.68 |
| *Rab3il1* | 6.47 | 0.68 | 2.24 |
| *Rb1* | 2.27 | 0.75 | 1.79 |
| *Rbm20* | 8.13 | 0.68 | 4.05 |
| *Rbm24* | 4.39 | 0.38 | 4.65 |
| *Rcor1* | 2.37 | 0.65 | 2.11 |
| *Rere* | 1.44 | 0.76 | 1.40 |
| *Rgs16* | 9.85 | 0.58 | 4.03 |
| *Rhoq* | 2.85 | 0.59 | 1.56 |
| *Rragd* | 6.99 | 0.60 | 2.86 |
| *Rreb1* | 2.95 | 0.73 | 2.90 |
| *Rybp* | 1.59 | 0.69 | 1.54 |
| *Ryr1* | 7.17 | 0.44 | 4.29 |
| *Ryr3* | 7.60 | 0.33 | 5.42 |
| *Sacs* | 8.99 | 0.36 | 4.54 |
| *Sbf2* | 2.40 | 0.75 | 1.50 |
| *Scn4a* | 44.46 | 0.41 | 22.12 |
| *Sdpr* | 8.32 | 0.52 | 2.33 |
| *Sema3d* | 14.09 | 0.33 | 4.50 |
| *Sema3e* | 2.82 | 0.46 | 1.54 |
| *Setd7* | 2.15 | 0.71 | 1.66 |
| *Sept11* | 2.21 | 0.75 | 1.63 |
| *Sh3rf1* | 1.55 | 0.75 | 1.61 |

| | Fold Change of Expression | | |
|---|---|---|---|
| ***Gene name*** | **Myo/Ad** | **Ad:siCTRL vs. siLsd1** | **Ad:Ctrl vs. LSD1(OE)** |
| *Shisa2* | 13.56 | 0.26 | 6.92 |
| *Shroom3* | 26.93 | 0.51 | 7.19 |
| *Slc6a6* | 4.23 | 0.61 | 1.80 |
| *Slc7a5* | 2.22 | 0.75 | 2.81 |
| *Smpx* | 9.96 | 0.51 | 9.97 |
| *Smyd1* | 24.43 | 0.31 | 11.55 |
| *Sntb1* | 10.10 | 0.60 | 7.00 |
| *Sorbs1* | 3.65 | 0.72 | 3.02 |
| *Sox4* | 2.42 | 0.64 | 1.88 |
| *Sptbn1* | 3.28 | 0.72 | 1.97 |
| *Srf* | 1.73 | 0.72 | 1.41 |
| *Srl* | 16.84 | 0.44 | 11.36 |
| *Srpk3* | 49.14 | 0.51 | 19.63 |
| *Stat3* | 1.62 | 0.77 | 1.65 |
| *Ston1* | 2.55 | 0.75 | 2.01 |
| *Strn* | 2.07 | 0.70 | 1.87 |
| *Sufu* | 1.45 | 0.71 | 1.63 |
| *Sync* | 4.50 | 0.77 | 1.81 |
| *Synpo2* | 11.01 | 0.61 | 4.73 |
| *Synpo2l* | 15.32 | 0.55 | 5.50 |
| *Tbc1d8* | 2.56 | 0.69 | 1.44 |
| *Tbx15* | 2.14 | 0.76 | 2.07 |
| *Tceal7* | 67.03 | 0.39 | 28.81 |
| *Tcf12* | 1.40 | 0.72 | 1.36 |
| *Tef* | 3.19 | 0.55 | 2.90 |
| *Thbs1* | 4.64 | 0.65 | 1.57 |
| *Tm6sf1* | 6.07 | 0.45 | 15.30 |
| *Tmem123* | 1.31 | 0.75 | 1.36 |
| *Tmem38b* | 2.29 | 0.74 | 2.12 |
| *Tmod1* | 3.12 | 0.67 | 3.25 |
| *Tnks* | 3.07 | 0.63 | 2.73 |
| *Tnnc1* | 6.02 | 0.56 | 5.10 |
| *Tnni1* | 9.41 | 0.50 | 6.56 |
| *Tnnt1* | 11.02 | 0.39 | 5.58 |
| *Tnnt3* | 7.06 | 0.55 | 4.75 |
| *Tnpo1* | 1.89 | 0.71 | 1.47 |
| *Tpm2* | 5.10 | 0.69 | 2.90 |
| *Trim55* | 15.66 | 0.55 | 5.77 |
| *Ttc3* | 1.96 | 0.74 | 1.52 |
| *Ttn* | 10.49 | 0.43 | 4.19 |
| *Unc45b* | 48.07 | 0.34 | 19.98 |
| *Utp14b* | 2.76 | 0.65 | 2.01 |
| *Utrn* | 4.39 | 0.70 | 2.15 |
| *Vash2* | 3.11 | 0.50 | 2.32 |
| *Vasp* | 3.33 | 0.74 | 2.34 |

| | Fold Change of Expression | | |
|---|---|---|---|
| ***Gene name*** | **Myo/Ad** | **Ad:siCTRL vs. siLsd1** | **Ad:Ctrl vs. LSD1(OE)** |
| *Vcl* | 3.46 | 0.77 | 1.56 |
| *Vps13c* | 3.52 | 0.72 | 2.59 |
| *Xpo4* | 1.52 | 0.72 | 1.68 |
| *Zbed6* | 4.92 | 0.71 | 2.75 |
| *Zbtb18* | 2.56 | 0.56 | 2.17 |
| *Zfhx4* | 1.46 | 0.74 | 1.60 |
| *Zfp516* | 1.73 | 0.75 | 1.69 |
| *Zfp568* | 2.12 | 0.68 | 1.82 |
| *Zim1* | 4.24 | 0.45 | 2.95 |

**Supplementary Table 2:** List of 74 inverselly regulated genes obtained from RNA-seq analyses in C2C12 cells at day 1 of adipogenic differentiation that are downregulated upon LSD1 overexpression and upregulated upon Lsd1 knock-down.

| | Fold Change of Expression | | |
|---|---|---|---|
| **Gene name** | **Myo/Ad** | **Ad:siCTRL vs. siLsd1** | **Ad:Ctrl vs. LSD1(OE)** |
| *0610007P14Rik* | 0.55 | 1.34 | 0.74 |
| *1700012B09Rik* | 0.24 | 1.94 | 0.29 |
| *9030619P08Rik* | 0.29 | 1.61 | 0.65 |
| *Ankle1* | 0.15 | 1.36 | 0.60 |
| *Camk2n2* | 0.22 | 1.45 | 0.69 |
| *Cchcr1* | 0.42 | 1.43 | 0.72 |
| *Ccl7* | 0.37 | 2.50 | 0.59 |
| *Cd53* | 0.55 | 2.32 | 0.40 |
| *Cdc7* | 0.20 | 1.41 | 0.67 |
| *Cenpu* | 0.22 | 1.38 | 0.62 |
| *Creg1* | 0.63 | 1.58 | 0.67 |
| *Crem* | 0.30 | 1.66 | 0.66 |
| *Cxcl1* | 0.12 | 1.65 | 0.58 |
| *Cxcl5* | 0.41 | 1.50 | 0.60 |
| *D430020J02Rik* | 0.28 | 1.38 | 0.62 |
| *Dhrs7b* | 0.38 | 1.47 | 0.65 |
| *Dnajc25* | 0.65 | 1.34 | 0.69 |
| *Dpysl2* | 0.47 | 1.33 | 0.69 |
| *Ecm1* | 0.72 | 1.90 | 0.75 |
| *Efnb2* | 0.41 | 1.32 | 0.44 |
| *Enho* | 0.61 | 1.95 | 0.49 |
| *Ephx2* | 0.00 | 2.13 | 0.47 |
| *Ereg* | 0.38 | 1.71 | 0.50 |
| *Fam132b* | 0.12 | 1.52 | 0.52 |
| *Fgl2* | 0.22 | 4.04 | 0.28 |
| *Glis1* | 0.07 | 3.41 | 0.41 |
| *Gm11974* | 0.46 | 1.34 | 0.54 |
| *Gpr137b* | 0.42 | 1.86 | 0.47 |
| *Gpr137b-ps* | 0.56 | 1.38 | 0.63 |
| *Has1* | 0.41 | 2.28 | 0.33 |
| *Havcr2* | 0.17 | 2.46 | 0.24 |
| *Hmga2* | 0.53 | 1.50 | 0.33 |
| *Htra1* | 0.67 | 1.69 | 0.51 |
| *Ier3* | 0.74 | 1.61 | 0.56 |
| *Iqck* | 0.26 | 1.41 | 0.62 |
| *Ly6a* | 0.26 | 1.83 | 0.54 |
| *Ly6c1* | 0.66 | 2.17 | 0.65 |
| *Mt1* | 0.03 | 1.54 | 0.53 |
| *Mt2* | 0.02 | 1.51 | 0.47 |
| *Nefl* | 0.51 | 1.62 | 0.45 |

| | Fold Change of Expression | | |
|---|---|---|---|
| **Gene name** | **Myo/Ad** | **Ad:siCTRL vs. siLsd1** | **Ad:Ctrl vs. LSD1(OE)** |
| *Nefm* | 0.59 | 1.86 | 0.49 |
| *Nmnat2* | 0.19 | 3.19 | 0.65 |
| *Npc2* | 0.51 | 1.51 | 0.70 |
| *Obfc1* | 0.60 | 1.33 | 0.68 |
| *Odc1* | 0.41 | 1.33 | 0.77 |
| *P4ha2* | 0.56 | 1.39 | 0.74 |
| *P4hb* | 0.55 | 1.36 | 0.77 |
| *Pdpn* | 0.31 | 1.71 | 0.44 |
| *Pidd1* | 0.24 | 1.32 | 0.67 |
| *Pla1a* | 0.44 | 2.28 | 0.47 |
| *Plaur* | 0.20 | 1.52 | 0.65 |
| *Plek* | 0.13 | 2.04 | 0.12 |
| *Prcp* | 0.17 | 1.43 | 0.64 |
| *Prg4* | 0.03 | 1.40 | 0.37 |
| *Prim1* | 0.18 | 1.35 | 0.54 |
| *Prl2c2* | 0.34 | 4.15 | 0.34 |
| *Prl2c3* | 0.48 | 2.48 | 0.35 |
| *Prl2c4* | 0.48 | 2.49 | 0.35 |
| *Ptgs1* | 0.70 | 1.37 | 0.56 |
| *Pxdc1* | 0.57 | 1.57 | 0.38 |
| *Slbp* | 0.25 | 1.46 | 0.67 |
| *Slc25a40* | 0.45 | 1.31 | 0.70 |
| *Slpi* | 0.12 | 2.20 | 0.18 |
| *Spc25* | 0.12 | 1.41 | 0.53 |
| *Srxn1* | 0.37 | 1.57 | 0.59 |
| *Them6* | 0.20 | 1.38 | 0.65 |
| *Timp1* | 0.70 | 1.85 | 0.68 |
| *Tmed5* | 0.57 | 1.87 | 0.64 |
| *Tmx2* | 0.40 | 1.44 | 0.69 |
| *Tpcn2* | 0.28 | 1.43 | 0.74 |
| *Ube2t* | 0.11 | 1.52 | 0.58 |
| *Vgf* | 0.02 | 2.89 | 0.24 |
| *Zfp367* | 0.37 | 1.68 | 0.60 |
| *Zfp948* | 0.24 | 1.63 | 0.46 |

**Supplementary Table 3:** Primers used for genotyping the mice

| Gene | Forward primer 5'-3' | Reverse primer 5'-3' |
|---|---|---|
| Ck-bAct-*LSD1* | AA TGCCTTCGAA TTCAGCAC | CCTTGTCA TCGTCGTCCTTG |
| Myf5-Cre | TTCCCGCAGAACCTGAAGATGTTCG | GGGTGTTATAAGCAATCCCCAGAAATGC |
| *Lsd1* WT/p | CCTCAGTAGGCCTGGTTTGT | TTGGTTTTGGTTGACCCTTC |
| *Lsd1* del | CCGTGGAAA TTCGTGCACTC | GCAGGCGGTTTGAAATGTATTC |
| Pax7-CreERT2 | ACTAGGCTCCACTCTGTCCTTC | GCAGATGTAGGGACATTCCAGTG |
| *Lsd1* (3xMut) | CCAGCTGCTTGTTGGTGC | TGGAGTGAAGTGGTTACCTGC |
| *GNZ* | TCATCTGCACCACTGGAAAG | GGGTCTTGTAGTTCCCGTCA |

**Supplementary Table 4:** Primers used for qRT-PCR analysis

| Gene | Forward primer 5'-3' | Reverse primer 5'-3' |
|---|---|---|
| *36b4* | GCGTCCTGGCA TTGTCTGT | GCAAA TGCAGA TGGA TCAGCC |
| *Hprt* | AGGGCATATCCAACAACAAACTT | GTTAAGCAGTACAGCCCCAAA |
| *mLsd1* | GTGTTCTGGGACCCAAGTGT | TAATGCCAGCAGCTTCTCCT |
| *hLsd1* | GCTCGGGGCTCTTATTCCTA | ATGTTCTCCCGCAAAGAAGA |
| *mGlis1* | CACACAGGCGAGAAACCATA | CTGTGTGCAGCTTCTTACGC |
| *hGlis1* | GCTGGAGAACCTCAAGATCC | GGTCTGTGTAGCGCTTGGAG |
| *Myod1* | TCCTCATAGCACAGGGGTGA | GCTCTGGCCAAGCAACTCTT |
| *Myog* | AAGTGGGGCTGTCCTGATGT | TAACAAGGGGGCTCTCTGGA |
| *Mef2c* | TCCTCTTATGCACTCACCCC | ATCAGACCGCCTGTGTTACC |
| *Ckm* | GCTTCGCGATAAGGAGACAC | AGCACATAGTTGGGGTCCAG |
| *Myh3* | TGATATCGCAGAATCGCAAG | AATTGTCAGGAGCCACGAAA |
| *Myl1* | CACCAATGCAGAGGTCAAGA | AAGACACGCAGACCCTCAAC |
| *Cebpα* | TTACAACAGGCCAGGTTTCC | CTCTGGGATGGATCGATTGT |
| *Fabp4* | GGGAACCTGGAAGCTTGTCT | TCGACTTTCCATCCCACTTC |
| *Adipoq* | GCACTGGCAAGTTCTACTGCAA | GTAGGTGAAGAGAACGGCCTTGT |
| *Pparγ* | GAAAGACAACGGACAAATCACC | GGGGGTGATATGTTTGAACTTG |
| *Prdm16* | CCCCCAACGCTCTCGGATCC | CCGAAGCAGCGGTTGCACAG |
| *Ucp1* | GTGAACCCGACAACTTCCGAA | TGCCAGGCAAGCTGAAACTC |
| *Elovl3* | ACTTCGAGACGTTTCAGGACTT | CCACTATGAGAAATGAGCTTACCC |
| *Cidea* | TGCTCTTCTGTATCGCCCAGT | GCCGTGTTAAGGAATCTGCTG |

**Supplementary Table 5:** Primers used for ChIP-qPCR analysis

| Gene | Forward primer 5'-3' | Reverse primer 5'-3' |
|---|---|---|
| *Glis1* | TGGGGCTACCCTAGATCCTT | GGGTTCATCCAGACGAGAGA |
| *Cebpα* | ATGCGTCCTTCAGACGAAGT | CTCTAGGGTCGCAGGTCAAG |
| *Ucp1* | TCAGGATTGGCCTCTACGAC | CACCCACATTGTCCATGAAG |
| *Adipoq* | GGCTTGCTTCTAGCCTTGTG | TGACCTCAGTCTGGCCTTCT |
| Unrelated | GTAGCTGTGACCACCCACCT | GACCCTGCATGAACTTTGGT |
